# Alarming coastal vulnerability of the deltaic and sandy beaches of North Africa

**DOI:** 10.1038/s41598-020-77926-x

**Published:** 2021-01-27

**Authors:** Abderraouf Hzami, Essam Heggy, Oula Amrouni, Gil Mahé, Mohamed Maanan, Saâdi Abdeljaouad

**Affiliations:** 1 Faculty of Sciences of Tunis, University of Tunis El Manar, Tunis, Tunisia; 2 Viterbi School of Engineering, University of Southern California (USC), 3737 Watt Way, Powell Hall of Engineering, Office 502, Los Angeles, CA 90089-1112 USA; 3 Jet Propulsion Laboratory, California Institute of Technology, Pasadena, CA USA; 4 National Institute of Marine Sciences and Technologies, University of Carthage, Tunis, Tunisia; 5 HydroSciences Laboratory, IRD, CNRS, University of Montpellier, Montpellier, France; 6 Institute of Geography and Planning, University of Nantes, Nantes, France

**Keywords:** Climate sciences, Environmental social sciences, Natural hazards, Ocean sciences

## Abstract

The arid coasts of North Africa, extending over 4633 km from the Gulf of Tunis to the Nile Delta, are undergoing pronounced shoreline retreats and coastal floodings that are reported as a consequence of the ongoing sea level rise resulting from global warming. Of particular interest are the abnormal shoreline dynamics for deltaic and sandy beaches, which are severely impacted by abrupt decadal variabilities in both climatic and anthropogenic drivers resulting in their increased vulnerability to disturbances from coastal hazards. Unfortunately, the evolution, distribution and impacts of these drivers remain largely unquantified, let alone understood, for these extensive arid coasts that harbor the major portion of North Africa’s population as well as unique and fragile marine ecosystems. To address this deficiency, we use GIS-based multi-criteria approaches combined with analytic hierarchy process to map the Coastal Vulnerability Index and the Socioeconomic Vulnerability Index along these coasts to investigate the amplitude and extent of shoreline deterioration resulting from sudden fluctuations in sediment transport to the coastline. We use the western bay of the Gulf of Tunis, the coasts of Tripoli and the Nile Delta as three validation sites for our vulnerability assessment. The statistical Integrated Coastal Vulnerability Index map reveals that 47% of arid North African coasts are characterized by high to very high vulnerability. In particular, we observe that the densely populated deltaic coasts in both Tunisia and Egypt are 70% more vulnerable than any others coast in the eastern Mediterranean Basin. These abnormally high-vulnerability extensive areas are also correlated with significant deterioration of coastal aquifers and hence in crop production, compromising local food security and resulting in increasing outflow migration trends. Both Tunisia and Egypt observed dramatic increases in the net population outflow migration by respectively 62% and 248% between 2000 and 2016, mostly from coastal areas. Our source analysis of the amplitude and extent of these high coastal vulnerabilities suggests that they result from the anthropogenic drivers of damming and rapid urban growth over the last few decades rather than the effects of global warming.

## Introduction

Aridification is globally sensed with an observable spread of desertic regions to coastal areas^1^ due to the changes in both multidecadal and interdecadal precipitation patterns^2^. Today, arid coasts represent an increasing portion of the world’s coastline Fig. 6A and harbor several high population urban areas. In particular, most drying trends in the last few decades are observed in Africa and Eurasia around the Eastern Mediterranean Basin^3^. This increased aridification is causing abrupt changes in coastal dynamics arising from variations in sedimentation and erosion regimes, mostly in sandy and deltaic shorelines^4^. Moreover, the precipitous urban growth and damming of rivers in these coastal arid areas is accentuating sandy beach erosion, causing abnormal retreat rates^5^. The population of the Mediterranean Basin grew from 276 M in 1970 to 522 M in 2019^6^. This represents an increase trend of 70% over the last fifty years and is set to rise to over 529 M by 2025^7^. This rapid population growth results in a high population density in coastal areas, especially in North Africa, leading to significant exploitation of the coastal resources and their subsequent deterioration^8–10^. Such pronounced and rapid degradations are materialized in widely observable shoreline retreats, increased coastal flooding and seawater intrusion in coastal aquifers^5,11^.

Several of these short and midterm coastal degradations resulting from anthropogenic factors such as urbanization and damming are often inaccurately attributed to sea level rise caused by global warming^12^. This interpretation of the causes of shoreline degradation in these arid areas of North Africa results in an ambiguous assessment of the distribution, amplitude and evolution of coastal vulnerability (physical and socioeconomic), leading to inadequate mitigation policies that only aggravate the damages. For instance, topographic analysis combined with sea-level rise modeling suggests that 1 to 3% of arid coasts extending along Tunisia, Libya, and Egypt are at high risk from future sea submersion by 2100 due to a mean sea level rise of 1.11 m under the RCP8.5 scenario in the IPCC fifth assessment report AR5^13^. The above will cause severe shoreline retreat by several tens of meters along extensive swaths of the Mediterranean shoreline^14,15^. Amrouni et al.^5^ suggests that such rates are already observable across extensive parts of the Tunisian coasts in the Gulf of Hammamet and that they are driven by environmental anthropogenic drivers rather than climatic ones. Even though the Mediterranean Basin consists of three fluctuating climatic regions—temperate, semi-arid and arid, which are characterized by changes in precipitation patterns—several localized environmental anthropogenic factors could have more potent and imminent impacts on coastal dynamics than long-term projected sea-level rise. In arid areas, urbanized coastal regions and notably sandy and deltaic beaches can exhibit severe sediment imbalance associated with the obstruction of sediment flow to the shoreline by man-made obstacles, such as dams and other water diversions and extensive concrete-covered areas, causing abnormal retreats along tens to hundreds of kilometers of the shoreline. The resulting observed localized land submersion can be confused with global sea-level rise that occurs on the continental scale.

The Mediterranean Basin is an area of particular interest in understanding coastal vulnerability, as half of its coastline is characterized by low-lying sedimentary coasts featuring beaches, dunes, reefs, lagoons, estuaries and deltas^16^. Moreover, both urban disaster zones and environmental hot spots in the Mediterranean Basin are already located disproportionately in low elevation coastal zones. The Low Elevation Coastal Zone (LECZ), defined by McGranahan et al.^17^ as the areas bordering the coast at that latitude, is less than 10 m above sea level and covers 2% of the world’s land area. In the Mediterranean Basin, the LECZ represent 20% of the total coasts^18^ and is the area of highest population concentration.

The sedimentary coasts bordering the Mediterranean Basin are mainly formed by terrestrial and marine-biogenic material. The terrestrial sediment discharged into the sea has been provided by the deltaic rivers throughout the last millennium^19^. The buildup of multiple dams in river catchments in recent decades, however, has significantly reduced the sediment transport of rivers to the coastlines,such is the case for both the Nile and the Medjerda Rivers in Egypt and Tunis, respectively^20–22^. Lacking their fluvial discharge supply, the downstream plains suffer from severe shoreline retreat and coastal aquifer salinization^5,11,23^.

Moreover, subsidence of coastal land, arising from sediment compaction due to building loads, harbor dredging, changes in coastal sediment supply, and subsurface resource extraction, increases the vulnerability to sea-level rise, storminess and exceptional river floods^24^. While artificial nourishment operations are widely deployed to compensate for the submerged areas, the newly created shorelines replacing natural ones have profound impacts on the conservation of marine ecosystems and species in coastal urban settings. Throughout the coastal areas of the Mediterranean basin, the marine ecosystem is increasingly affected by the rise in climate aridity, changes in land use, pollution and declining biodiversity^25^. Simulations for the RCP4.5 and RCP8.5 scenarios by Guiot and Cramer^26^ suggest significant changes in the wetlands and vegetation coverages for these areas during the present century inducing desert expansion toward the North African coasts and the in-land regression of alpine forests in southern Europe.

A longer observation period along with high-resolution sediment budget studies are necessary to quantify the extent to which continued trapping of sediment behind dams is impacting the overall deltas’ stabilities in the Mediterranean Basin^19^.

For all of the above, North African coasts face significant vulnerabilities that are even more accentuated by the increasing occurrence of coastal hazards such as floods and wave storms^27^ as well as tsunamis^28^, which are continuously causing severe economic damage such as the recent coastal floods observed in Alexandria following the massive “Dragon Storm” depression in March of 2020, which had wave height values exceeding 5 m as observed from the northwest^29^.

It is important to note that the arid North African coasts located in the central and eastern Mediterranean Basin, extending over 4633 km from the Gulf of Tunis to the Nile Delta, are among the world’s regions of highest population density and growth for the last few decades^30^.

**Figure 1:**
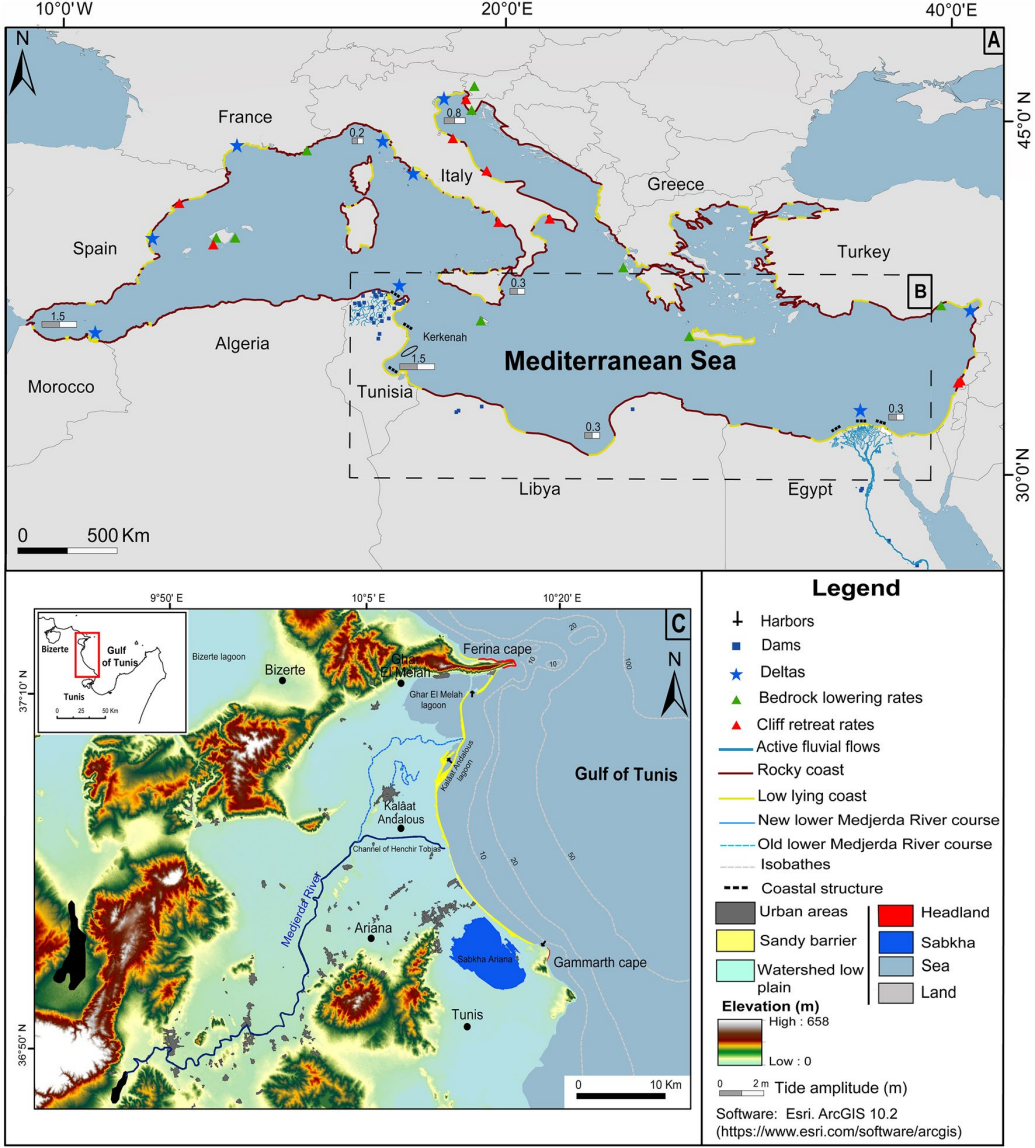
(**A**) Contextual map of our study area; the arid North African coast mainly constituted of (**B**) deltaic plains and sandy shores and (**C**) the location and characteristics of our main study site in the Gulf of Tunis. The geomorphological characteristics and the tidal amplitude information of the Mediterranean coast are based on data from^82^. The coastal characteristics of both Tunisia and Egypt are based on data from APAL^83^ and Frihy and El-Sayed^84^.

To assess the origin, amplitude and extent of coastal vulnerabilities, we generate maps of the Coastal Vulnerability Index (CVI) and the Socioeconomic Vulnerability Index (SVI) for coasts on both sides of the Mediterranean Basin Fig. 1A to benchmark the vulnerability of North African coasts Fig. 1B against other areas of the basin. In our study, we define physical vulnerability as the response of the coast to sea-related natural hazards such as tsunamis, sea level rise, submersion and wave storminess. Socioeconomic vulnerability refers to the social and economic conditions of a given population that determine its resilience and ability to cope with or adapt to the coastal hazards as summarized above^31,32^.

We use the western bay of the Gulf of Tunis (WBGT) Fig. 1C as a primary study site in addition to two other secondary sites along the coasts of Tripoli and the Nile Delta to assess the vulnerability of the low latitude deltaic zones to shorelines retreat. We use GIS-based multi-criteria approaches and the combination of CVI and SVI indices within the analytic hierarchy process (AHP) integrated within the statistical Integrated Coastal Vulnerability Index (ICVI) to generate vulnerability maps of the arid North African coasts located in the central and eastern parts of the Mediterranean Basin. The primary objective of mapping the different vulnerability indices is to provide a single metric that reflects several physical and economic parameters assimilated from different datasets in order to identify zones with high vulnerability and to assess proper mitigation strategies^33^. In light of the origins, evolution and extent of these vulnerabilities, we discuss the socioeconomic implications of these different coastal indices, most notably for food security and migration fluxes in the southern part of the Mediterranean Basin. The study sites and methods are detailed in the supplementary material.

## Results

Taking into account the physical and socioeconomic drivers in North Africa’s Mediterranean shores, we first assess the large-scale vulnerability of the whole coast, and then we focus on the key areas of the Gulf of Tunis in Tunisia, the shores of the Nile Delta in Egypt, and the Tripoli coast in Libya.

### Large-scale vulnerabilities along the North African coast

Our assessment of the vulnerabilities along all the arid deltaic and sandy beaches of the North African coast is performed by computing the geographical distribution of three parameters: (1) the coastal vulnerability index (CVI), (2) the socioeconomic vulnerability index (SVI) and (3) the Integrated Coastal Vulnerability Index (ICVI) as follows:

#### Coastal vulnerability index (CVI)

**Figure 2:**
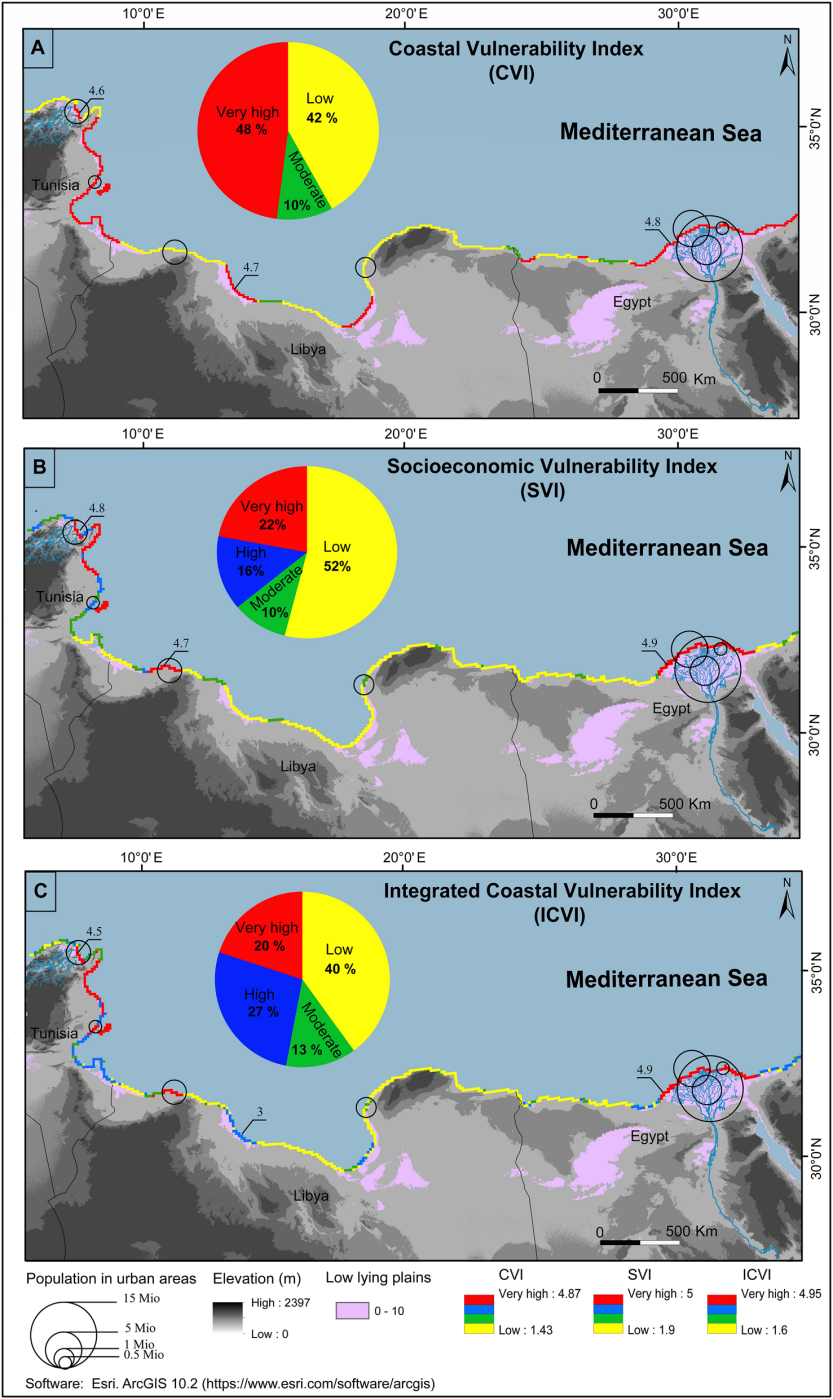
Vulnerability scale for the three indices characterizing the risk to immersion in the North African coasts. (**A**) Coastal Vulnerability Index (CVI); (**B**) Socioeconomic Vulnerability Index (SVI) and (**C**) Integrated Coastal Vulnerability Index (ICVI). Population number in urban areas are derived from CIESIN^85^.

The CVI map of North African arid coasts Fig. 2A shows the extent of vulnerabilities in coastal zones. The physical-based CVI values range from 1.43 to 4.87. Gornitz^34^ classify vulnerability ratings according to four classes of risks: low (1.43 to 2.29), moderate (2.29 to 3.15), high (3.15 to 4.01), and very high-risk areas (4.01 to 4.87).

As shown in Fig. 2A, 48% of the North African coast presents very high risk values. From the center of the Mediterranean Basin to its eastern coasts, the CVI spatial distribution suggests that the very highly vulnerable areas are located along the northeastern Tunisian coast, with values ranging from 4.6 to 4.7 along the Gulf of Tunis and Gulf of Hammamet coasts and the Kerkennah Islands. Along the central Libyan coast, CVI values ranged from 4.7 to 4.8 along the Gulf of Sirte. Along the deltaic Egyptian coastal plain, CVI values ranged from 4.8 to 4.9 on the coasts of Alexandria.

These CVI classifications suggest that ~ 10% of the investigated coastal areas are moderately vulnerable and 42% are characterized by a low vulnerability index. Those areas with lowest vulnerability are located along the northwestern Tunisian coast with CVI values ranging between 1.9 and 2, along the eastern Libyan coast with a CVI range between 1.6 and 1.7, and along the far west coast of Egypt where values range between 1.8 and 1.9.

#### Socioeconomic vulnerability index (SVI)

Figure 2B shows the socioeconomic vulnerability index map for the whole Mediterranean Basin along 18789 km of coastlines. A calculated SVI value from socioeconomic variables has been classified as a dimensionless “risk” variable between 1 and 5 according to the ranks of vulnerability established by Gornitz^34^: low risk (1.9 to 2.67), moderate risk (2.67 to 3.44), high risk (3.44 to 4.21), and very high risk coasts (4.21 to 5).

Mapping of SVI values suggest the following classifications: 22% of the shore is at very high vulnerability, 16% is at high vulnerability, 10% is at moderate vulnerability and 52% of the shore falls under low risk coasts. The highest SVI values are located along coastal urbanized cities such as Ariana (Tunisia) with an average score of 4.8 to 4.9, in Tripoli (Libya) with score values between 4.7 and 4.8 and in Alexandria (Egypt) with a score of 4.8 to 4.9. The Kerkennah Islands located east of the study areas are also marked by a very high risk score ranging between 4.4 and 4.5. The spatial variability map shows that low risk areas are mostly located along the Libyan and the northwest Egyptian coasts with values between 1.9 and 2.4. Only a few hundred kilometres of Tunisian coast falls under moderate risk, with an average score between 2.3 and 2.4.

#### Integrated coastal vulnerability index (ICVI)

The Integrated Coastal Vulnerability Index (ICVI) is calculated from from Eq. 5 (as described in the supplementary material in section 3.3 (Analytical hierarchical process (AHP)) using the physical-based index CVI and the socioeconomic index SVI. The resulting ICVI values are georeferenced and integrated into the ArcGIS database to generate coastal vulnerability maps along the North African coast as illustrated in Fig. 2C. ICVI scores range from 1.6 to 4.95, corresponding to the lowest and highest risks, respectively. The total range of ICVI scores is divided into a scale of four equal classes, where each identifies a given risk level for coastal hazards: a very high score (4.13 to 4.95), high score (4.13 to 3.23), moderate score (2.4 to 3.23) and low range (1.6 to 2.4).

Figure 2C suggests that 20% of the North African coast can be classified as a very high-risk area and 27% classified as high risk. The very high to high vulnerability zones extend along northeastern Tunisian and Egyptian coasts. Coasts showing very high-risk vulnerability scores are located in the Gulf of Tunis and the Nile Delta. In particular, the Medjerda delta and the Gulf of Hammamet have ICVI values between 4.3 and 4.6, and in Alexandria we observe values ranging between 4.85 and 4.9 along the sandy barrier of the Lake Burullus. Areas of very high to high vulnerability represent 47% of the North African coast, 71% of the total Tunisian coast, 9% of the Libyan coasts and 70% of the total Egyptian coasts on the Mediterranean Sea. The Kerkennah Island coasts in Tunisia are all classified as very high-risk zones (100% of the 174 km of coastline) with ICVI values ranging from 4.5 to 4.6 Fig. 2C.

The southeastern Tunisian coast, Libyan coasts and western ridge of the Nile Delta coast show moderate to low-risk vulnerability with values of ICVI ranging between 1 and 2. These moderate to low risk vulnerability areas represent respectively 13% and 40% of the total coastline in our study area as shown in Fig. 2C.

### Localized vulnerability trends in the Gulf of Tunis

The variables used in our localized coastal vulnerability assessment along the WBGT are divided into three major categories: geological, physical and demographic (Table S3). The implementation of these data sets in our vulnerability calculation reveals substantial spatial variability of the vulnerability risk in the WBGT (Supplementary data: Fig. S1A,B). Among the variables considered in our analysis, the coastal elevation and the mean tide range which are primarily indicators of coastal vulnerability risk to submersion and coastal flooding (Supplementary data: Fig. S1A). The shoreline retreat is observed along all the northern part of the Gulf of Tunis with localized very high-risk vulnerability hot spot (Supplementary data: Fig. S1A). The socioeconomic assessment of the embayed coast of the WBGT shows high-risk scores of the land use, settlement and road network variables NE and south of the study area (Supplementary data: Fig. S1B). The other indicators are low to moderate-risk scores.

**Figure 3:**
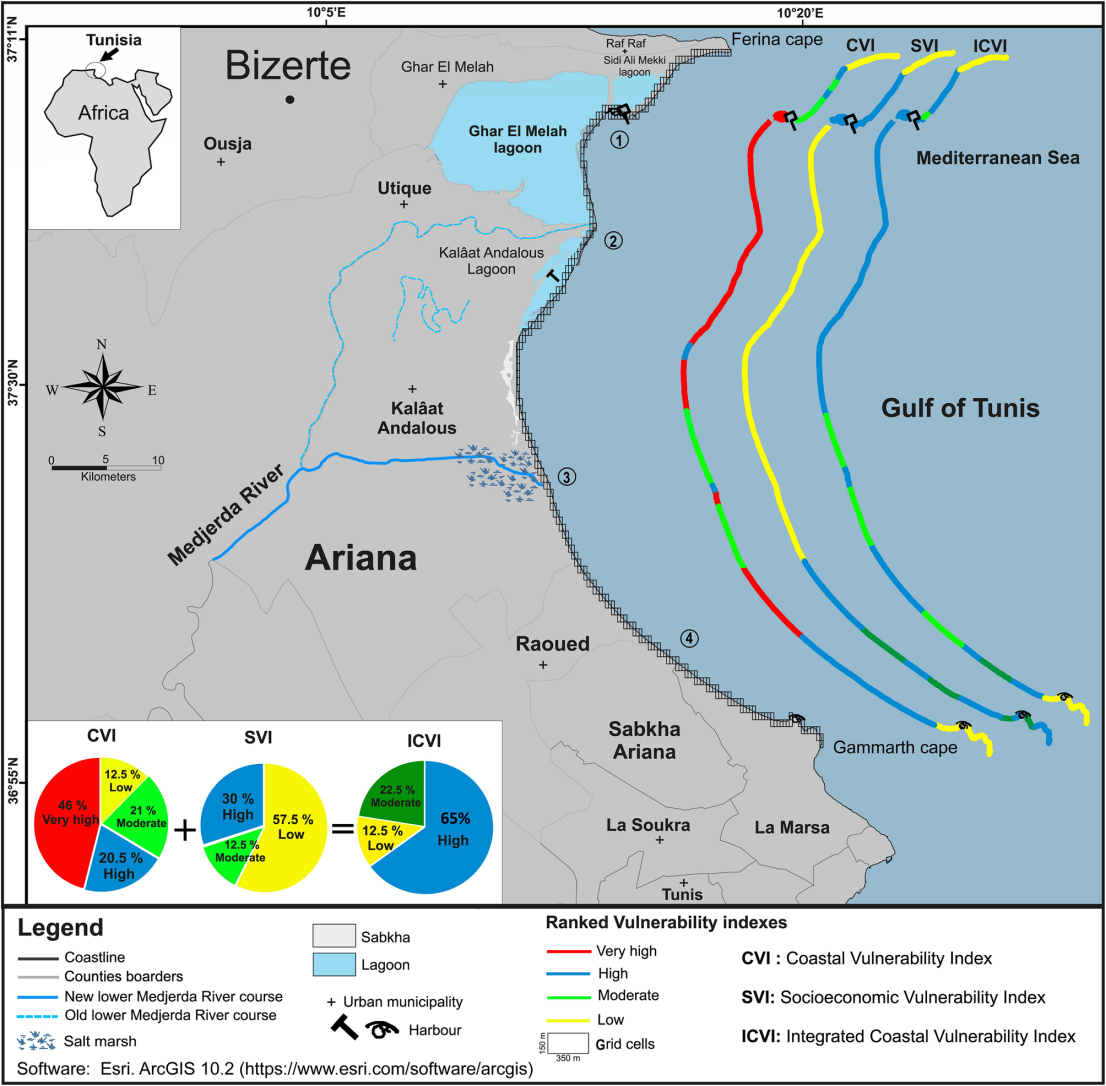
Vulnerability scale of physical and socioeconomic variables CVI, SVI and ICVI for the primary study site of the Gulf of Tunis.

We subdivide this localized study zone into four areas as shown in the Fig. 3, in order to assess the vulnerability trends and their associated variables: (1) the area extending from the north to the south of the bay along the Ghar El Melah beaches, (2) the area bordered by the old Medjerda River course (south of the Ghar El Melah Lagoon and covering the Kalâat El Andalous sandy barrier), (3) the area aligned with the new lower Medjerda River course’s deltaic plain, and (4) the area south of the WBGT along the Raoued coasts.

The multi vulnerability indicators CVI, SVI and ICVI of the WBGT are mapped in the Fig. 3. The CVI spatial vulnerability distribution map of the coast reveals 15 segments with a unique identity number in its corresponding attribute table. The SVI stretch is subdivided to 11 segments and the ICVI is divided in 13 segments.

#### Physical drivers

The physical-based CVI values are between 1.52 and 4.75. According to these values, different vulnerability areas were delimited in four classes: low (1.52 to 2.33), moderate (2.33 to 3.13), high (3.13 to 3.93), and very high risk (3.93 to 4.75). A total of 46% of the WBGT coastline has very high-risk values along areas 1, 2 and 3 (red CVI segments in Fig. 3. About 20.5% of the coast is high-risk areas (blue CVI segments in Fig. 3, including most of area 4 and a few meters of beaches in the northern part of area 1 and in the southern part of area 2.

The long-term shoreline movement monitoring calculated by the Digital Shoreline Analysis System along areas 1, 2, 3 and 4 during the years 1882 (4 m), 1936 (2 m), 1974 (2 m), 1988 (20 m), 1999 (20 m) and 2016 (10 m) reveals spatial and temporal variabilities shoreline evolution as mentioned in Supplementary data Fig. S2 and Table S8. In this case, an automatic shoreline detection method using photogrammetric image classification and manual digitization is developed and is fully detailed in^5^.

At the beginning of the twentieth century, area 1 shows positive trend in the End Point Rate (EPR) values between 1882–1936 and 1974 of 1.85 to 3.82 m/year (± 0.16 to 0.27). The erosion trend started from the period of 1988–1999 and 2016 where the EPR values reveals a regression of − 2.39 m/year (± 0.2) to − 5.23 m/year (± 0.2) and an average of Net Shoreline Movement (NSM) values of − 26.37 to − 88.95 m (± 2 to 3.7) respectively (Supplementary data Fig. S2).

The area 2 shows a positive shoreline evolution trend from 1882 to 1936 with an average of NSM of 154.78 m (± 14) but the erosion trend stated since 1936 to 2016 with an increasing trend of the shoreline retreat of EPR of − 11.61 m/year (± 0.16) during the period between 1936 to 1974 to − 32.64 m/year (± 0.2) during the period 1988 to 1999. The highest NSM values are observed along the area 2 with losses of − 1571.88 m (± 6) between 1936 and 1974 (Supplementary data: Table S8).

The area 3 is characterized by a positive shoreline movement rate during the years 1936, 1974 and 1988 with an average of EPR value of 4.33 to 16.72 m/year (± 0.16 to 0.18) (Supplementary data Fig. S2). The erosion trend along the area 3 started since 1988 with a maximum sediment loss of NSM of − 219.13 m (± 2) to 1999. The NSM shows the negative movement values during the last decades between 1999 and 2016, with an average of − 47.69 m (± 3.7).

In the area 4, the early twentieth century period shows a stable shoreline trend with an EPR values of − 0.10 m/year (± 0.27) between 1982 and 1936 and with an EPR values of 8.43 to 2.26 m/year (± 0.18 to 0.2) respectively from 1974–1988–1999. Moreover, the positive shoreline evolution trend is interrupted by a negative period between 1936–1974 when the coast loss is calculated with an average of EPR of − 3.13 m/year (± 0.16) and the NSM values of − 118.82 m (± 6). The fast-urban growth period from 1999 to 2016 experienced an erosion rate with an EPR value of − 1.58 m/year (± 0.22) (Supplementary data Table S8; Fig. S2).

#### Socioeconomic drivers

For the SVI values the different risk levels range between 2.8 and 4. These ranges are divided into three equal vulnerability risks levels: low (2.8–3.2), moderate (3.2–3.6) and high (3.6–4).The socioeconomic vulnerability variables are in the highly-risk ranges for about 30% of the total Gulf of Tunis as shown in supplementary data (Fig. S1B). The high-risk areas are located in the middle part of the area 1 and along the area 4.The lowest range of values indicates that 57.5% of the coast is at low risk areas along the southern area 1, the area 2 and 3 Fig. 3.Areas with moderate risk level constitute 12.5% of the WBGT coast, in particular in the northern and southern parts of the bay in localized beaches.

Our results suggest that the ICVI index along the WBGT arid coast varies between 1.5 and 4.68. The ranges are divided into three levels: low-risk (1.5–2.56), moderate-risk (2.56–3.62) and high-risk (3.7–4.68) levels.

The ICVI spatial distribution scheme indicates that 65% (~ 26 km) of the WBGT coastal ridge falls under high-risk vulnerability to the rise of sea level. It is mostly located from north to southward on the area 1, 2 and 4. Some localized high-risk zone is present in a several hundred meters in the area 3. About 22.5% (~ 9 km) are characterized by moderate-risk areas mostly located in areas 3 and 4. The lowest scores of ICVI, which represent 12.5% (~ 5 km) of the total coastal length, are located on rocky promontories.

## Origins and implications of the coastal vulnerability

Global warming is expected to significantly increase coastal vulnerability due to potential changes in precipitation patterns, floods and of marine storms intensities^35^. Our results suggest that the arid deltaic and sandy beaches of the North African coasts are among the most vulnerable to disturbances from coastal hazards in the central and eastern Mediterranean Basin. Their vulnerability is being increasingly observed during mild to severe weather events that are occurring along these extensive coastlines. For instance, in northeast Tunisia, the Nabeul large flash flood of September 2018 and the Ariana flood in October 2019 brought an average of 90 to 220 mm of rainfall in one hour, respectively, causing both material damages and human losses^36^. Moreover, on October 2019, the Mediterranean coastal areas of Egypt were ravaged by an extremely uncommon “medicane” bringing tropical-like storm-force winds, heavy rainfall and coastal flooding causing severe material and human losses. Since 1980, Mediterranean Sea surface temperatures have increased between 1 and 2 degrees^37^. The warmer sea surface temperature in the eastern part of the Mediterranean Basin is suggested to be the catalyst for such atypical medicane-type storms^38^. An increased occurrence of medicanes in the western Mediterranean Basin and in the Black Sea is projected and could be associated with a reduction of storm tracks in adjoining areas, particularly in the central Mediterranean^37^. Moreover, future extreme wind events reaching speed up to 60 kt are expected to become more frequent in all Mediterranean sub-basins^37^. As these repeatedly extreme and unusual phenomena are occurring along highly vulnerable coasts, more urban damage and environmental impacts are being sensed.

### Physical drivers of coastal vulnerability

As mentioned in “Sea level rise, tides and waves” section, the highest values for the different indices of coastal vulnerabilities are observed along the eastern coasts of Tunisia (including the coasts of Kerkennah islands) and along the Gulf of Sirte in the central Libyan coast to the Nile Deltaic coastal plain as shown in Fig. 6A, B. The observed vulnerabilities can be attributed to three physical factors and a socioeconomic one as discussed below:

#### Coastal topography

The very high and high vulnerability areas in the North African arid coasts represent 71% of the total Tunisian coast, and 19% of the Libyan coast and 72% of the total Mediterranean Egyptian coast. This high vulnerability values can be partially attributed to the geomorphology of the North African arid coasts where more than its half consist of a sandy ridges, lagoon and salt marsh deposit and alluvial deltaic plain (Table S1). The most vulnerable areas are located in the Low Elevation Coastal Zone (LECZ) of deltaic-plain River such as the Medjerda and the Nile Fig. 2, which includes alluvial plains and coastal lagoons that are almost flat and close to sea level. The low topography of the coastal landforms and the low slope of the beaches represent a priority factor influencing to the high CVI^39^. It explains the very high-risk rate observed over the total coastline of the Kerkennah Island.

#### Geological and geomorphological settings

The North African arid coasts are characterized by mixed siliciclastic-carbonate sediments from the tertiary and quaternary (i.e. Pleistocene and Tyrrhenian) outcrops^40–42^. The low-lying plains are filled by alluvial washes and rivers deposits. Moreover, the geological setting and related tectonic movement showed non-negligible subsidence phenomena in those continental shelves such as the Nile Delta coastal areas. As a result of the complex tectonic setting of the Eastern Mediterranean, earthquakes occurred in the vicinity of the Egyptian continental margin both in recent and historical times^43,44^. Rates of subsidence across the Rosetta and Damietta promontories are significant, ranging from 2.5 to 3.5 mm/year^45^. Similarly, the Medjerda plain undergo a subsidence rate of 10 mm/year^46^ (Table S1). The calculated physical indices according to 

Gornitz^47^ represent a minimum threshold of vulnerability. The highest present-day subsidence rate in high population density areas increase the physical risk vulnerability rating. Physical risk vulnerability herein defined as the geological and geomorphological resistance to disturbances caused by sea-related natural hazards such as tsunamis, sea level rise, submersion and wave storminess.

#### Sea level rise, tides and waves

The multi-Scale Coastal Vulnerability Index adopted by Thieler and Hammar-Klose^48^ classifies the Sea Level Rise as the fifth prior vector factor with 5%. This physical factor is undergoing an increasing rate since the last decades with 3.2 mm/year^49^. The coasts of southern Tunisia and the northeastern Egyptian ones show respectively 4.3 and 4.9 mm/year rates for the sea level rise (Table S1). The associated risks to these SLR observations are increased flooding, accelerated landward saline intrusion, storm surges and shoreline erosion^50–52^.

**Figure 4:**
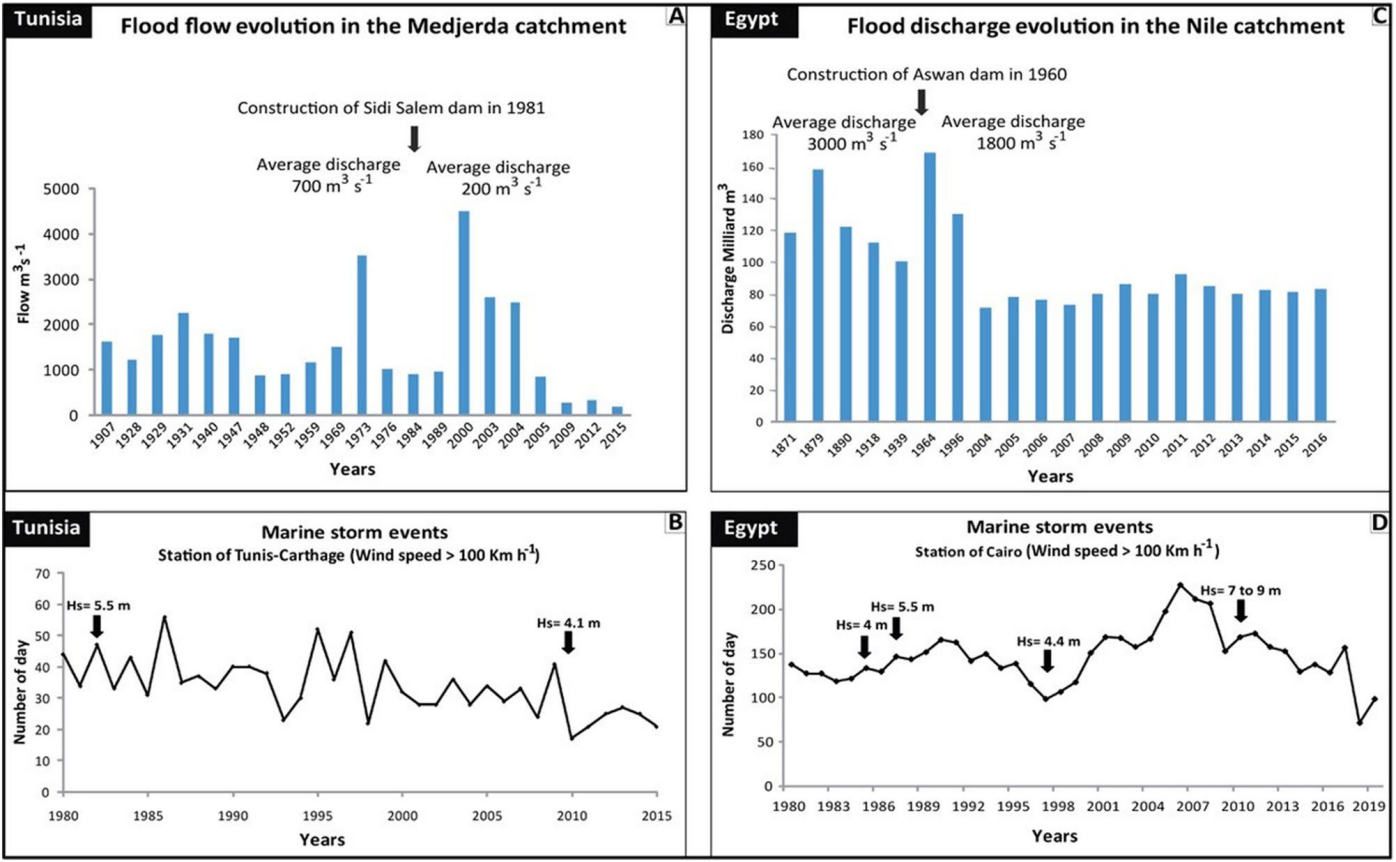
(**A**) Temporal evolution of the flooding events in the Medjerda River and (**C**) Nile catchments, for the last century; (**B**) Marine storms occurrence from 1980 to 2020 in the WBGT and (**D**) Nile Delta. Statistical data are collected in the stations of Tunis Carthage (Tunisia) and Cairo (Egypt). The significant wave height was established from the data of the historical records along the Tunisian^46^ and the Nile Delta Coasts^56^.

The tidal range and the significant wave height are not a majorly weighted variable, as the Mediterranean Basin is a semi-closed sea with a limited wave fetch and under microtidal regime (tides < 2 m) Fig. 1A. The global warming impact is to increases the occurrence and intensity of storm surges^25,53^. Alternatively, the METEOCEAN Model studies (e.g. Sartini et al. ^54^) suggest that the Mediterranean storm-waves characteristics can also significantly affected by seasonality arising from weather perturbation regimes occurring during different seasons. Several extreme sea storms have recently occurred in Rosetta promontory in Abu-Qir Bay in Egypt Fig. 4D with a maximum value of significant wave height of 4.19 m with a wave period of 10.7 s observed in November 1986 from northwesterly direction^55^. According to Iskander^56^, measured wave records along the Nile Delta coasts from 1985 to 2010 reveals increases in sea wave height trend by a rate ranging from 2.6 to 2.9 cm/year. Moreover, in the northeastern Tunisian coasts, the incident sea waves height records reach 5.5 m in winter from the Northeast Fig. 4B. The geomorphology of the North African arid coasts reveals a succession of drift and embayed swash beaches shaped by waves arriving obliquely and parallel to the shore respectively,mainly supplied by both terrestrial material and offshore supplies Fig. 1A. Thus, the coastal sediment budget equilibrium is depending of the terrestrial and net offshore supply, which are disturbed by damming and urban growth^5^. Hence, the negative sediment unbalance arising from increased occurrence of coastal hazardous weather events combined with sediment trapping endanger beach stability causing the alarming shoreline erosion and is responsible of the high coastal vulnerability observed near urban areas.

Moreover, the damming of rivers obstructs sediments transports delivery to the coastal zones, depriving the ecosystems from the minerals and nutrients they carry. Maavara et al.^57^ ecological model prediction for the midcentury, suggests that more than half of the worldwide rivers flowing to the sea will experience greater removal of silicon over nitrogen and phosphorus, as a consequence of increasing constructions of hydroelectric dams. The above have an impact on the role of diatoms in nearshore marine production, as they are increasingly outcompeted by other, potentially harmful, algae that do not need silicon to grow^57^.

#### Shoreline multi-decadal dynamic

Shoreline evolution is the physical variable considered as a crucial visual indicator of the coastal vulnerability to natural hazards. The natural and anthropogenic drivers control the temporal trends of the shoreline dynamic. Our localized CVI assessment along the Tunisian coasts suggests that the highest vulnerable classes extend over 46% of the WBGT along the areas 1, 2, 3 and 4 (supplementary material: Fig. S1A,B). Those areas are also the most eroded with a maximum NSM negative value during the last multi-decades period (Supplementary material, Table S8).

The long-term DSAS evolution suggests two evolution trends of the embayed sedimentary and deltaic plain areas: (1) the natural dynamic and (2) the anthropogenic one.

The natural response at the beginning of the twentieth century shows a positive evolution of the gulf of Tunis’s sandy beaches. The active yield discharge of the old Medjerda River course associated with the offshore bioclastic materials supplied the coasts and the shores are accreting with an accretion rate > 1 m/year. Even so, we observe an erosion trend of the southern bay stretch (area 4) between the period of 1936 to 1974 when the coast loss is calculated with an average of EPR of − 3.13 m/year (± 0.16) and the NSM values of − 118.82 m (± 6). During the early 1900s, several droughts and irregularity in the rainfall’s periods have been recorded which have diminished the sediment discharge fluxes of the Medjerda River such as the flood of the upstream Delta occurring in March 1973 with a maximum water flow of 3500 m^3^/s Fig. 4A.

The coastal sandy barrier in the area 4 is bordering the arid paralic sedimentary system of the Ariana’ sabkha Fig. 1C. It has been a closed lagoon from 1300 to 2200 BP^58^ and is filled by the wave reworking action of the NE migration of the Medjerda terrestrial flume associated with subsidence due to local Quaternary tectonic movement associated with NW–SE normal fault and isostatic post-glacial fluctuation due to hydro-climatic factors^59^.

The shoreline evolution during the last few decades is characterized by severe erosion trend, especially at the area 2 with a value of EPR up to − 10 ± 0.2 m/year. Hzami et al.^60^, report similar observation along this coastal area with maximum rates of retreat by − 20 ± 0.15 m/year.

**Figure 5:**
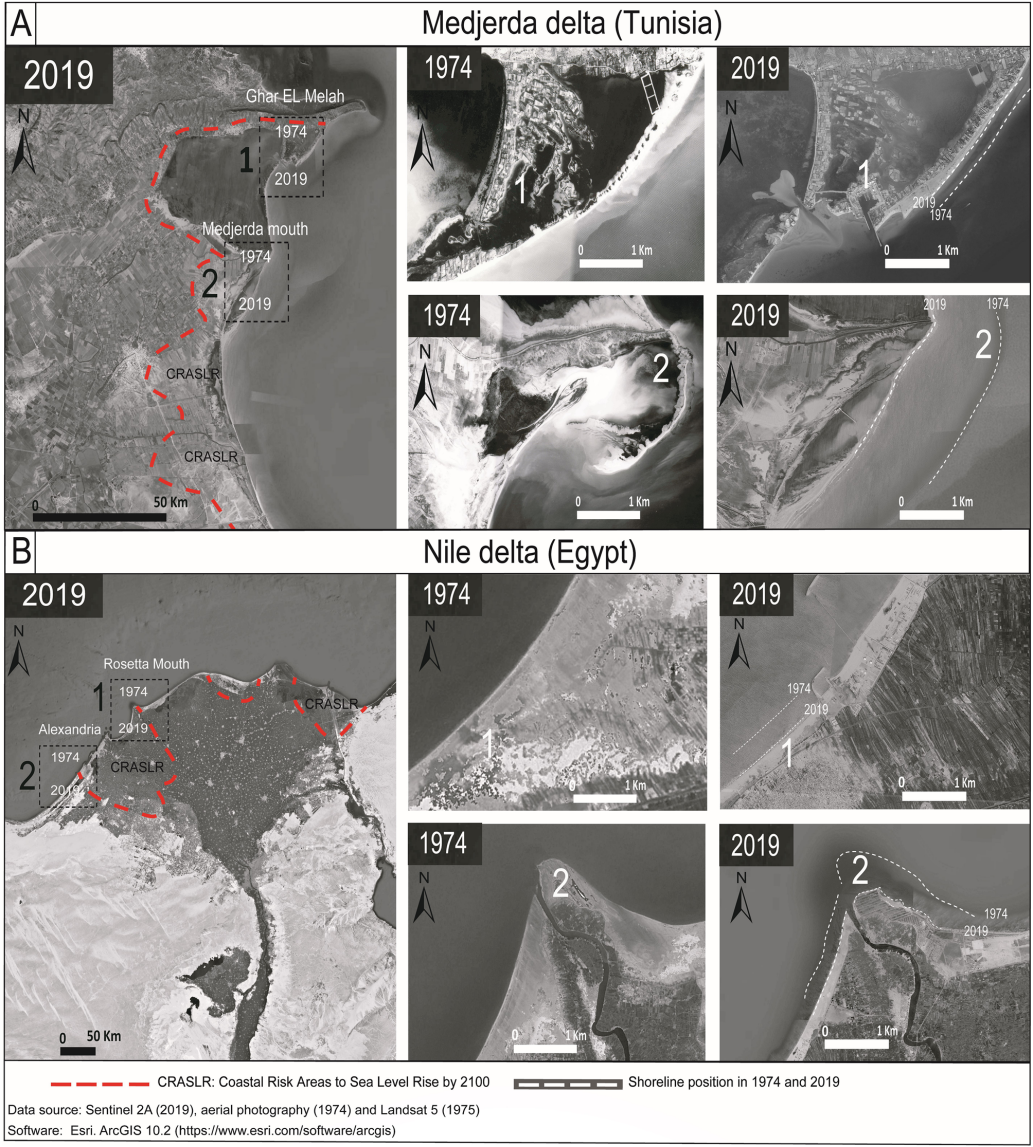
Observed shoreline retreat and deterioration between 1974 and 2019 in the coasts of (**A**) Medjerda and (**B**) Nile River delta plain. The numbers indicate the evolution of key features in the land coverage in Medjerda Delta (**A**) (1) indicate the change in wetlands evolution transforming cultivate wetlands to salt marsh-lakes landforms and (2) indicates the alarming reduction in sandy spit areas; in Nile Delta (**B**) (1) indicates the natural landforms classes disappearance and replaced by the land use occupation and cultivated crops and (2) The critical erosion of the Rosetta Mouth. Red dotted line refers to the limitation of the Coastal RiskAreas to Sea Level Rise by 2100 according to Kulp et al.^86^.

The alarming NSM values recorded along the area 2 with a maximum value of − 1571.88 m (± 6 m) between 1936 to 2016 is related to the anthropogenic drivers. Moreover, the deviation of the natural course of the Medjerda River since 1936 southward, the establishment of port dikes since 1974 and the dam building in 1982 have all considerably reduced and disturbed the sediment discharge to the coast^21^ Fig. 5A. The shoreline retreat accentuates the coastal vulnerability of the low-lying less-supplied areas.

Similarly, the shoreline retreat and the reduction in sand supply in the coastline of the Nile Delta are attributed to the construction of the Aswan High Dam in 1960s^61^. The high erosion rate in the Nile Delta coasts between 1955 and 1983 is − 114 m/year^62^^,^ caused by sediment trapping as a consequence of the river damming^61,63^ (Fig. 4C) and the physical imbalance of the coastal dynamics caused by maritime structures Fig. 5B. Both of these factors contribute to the very high-risk scores of vulnerabilities observed along the Nile Delta front and prodelta deposits. The low coastal vulnerability risk for Libyan coasts is attributed to balance in coastal sediment budget due to the absence permanent network of flowing rivers feeding the shores and the relatively higher coastal slopes.

Flows replenishing the coastlines in the Mediterranean basin are continuously being degraded in term of water quality due natural drivers such as lower precipitations and heatwaves as well as anthropogenic drivers such as damming, pollution and overexploitation. This degradation is more accentuated in the southern part of the Mediterranean basin where coastal wetlands and nearshore marine ecosystems hosting fishery resources are observing lower productivity arising from degradation of flow quality and its depletion from several essential mineral and nutritive^25^. The above have severe impacts on both agriculture, fisheries and aquaculture, hence compromising the food security and the economical sustainability of the most arid parts of the Mediterranean coastline that is already suffering from overfishing and abrupt growth in coastal urban development^64^.

### Urban growth in coastal areas

In our investigation, the socioeconomic variables are derived from the population density, coastal land-use, road networks and settlements. The SVI scores helps assess the vulnerability of the coastal population as more than 30% of the population of the North African arid coastlines in coastal urban areas. In the socioeconomic vulnerability map Fig. 2B, we note that the very higher SVI values are located around areas with high urban growth (Table S1). According the pairwise comparison of the socioeconomic variable (Table S5) the population density is a priority parameter per 60% than other variables as coastal land-use, road networks and settlements.

In Tunisia, the socioeconomic vulnerability index reveals very high scores of 4.8 and 4.9. The Tunisian population growth rate is about 1.03% over the last decades (between 2004 and 2014), the average population density is about 700 persons/km^2^ and the urbanization rate are increasing by 65%^65^.The increasing population density located in the coast of the gulf of Tunis’s during the last decades (2004–2014) is about 87% (Table S1).

The shores in Egypt show the same high socioeconomic vulnerability ratings, with a score of 4.8 to 4.9. The Egyptian population growth rate over the last decade is estimated to average above 2%, with development that is dramatically urban close to 50%^66^. In the Nile Delta, the population density averages 1000 persons/km^2^ with a growth rate of 62.6% along the Nile Delta over the 2005 to 2017 period as shown in Table S1.

The highly urbanized areas in the Libyan coasts such as the coasts of the city of Tripoli experiment a very high social vulnerability rating between 4.7 and 4.8. The Libyan population growth rate is estimated to be 1.45%, with development that is dramatically urban ~ 80%^66^ The average population density in Tripoli is about 200 persons/km^2^.

Our regional assessment for the coastal vulnerability over the North African arid coasts that is mostly constituted of sandy beaches is consistent with the results of the Global Projection Model of Coastal Vulnerability (GPMCV) in^67^, which estimated the potential disappearance of 50% of the global distribution of sandy beaches in the low elevation coastal zone by 2050. Even though the North African sandy coasts were not considered in the GPMCV analysis, due to the lack of publicly available data for the area to the investigators, our study here in address this deficiency and confirm that the extent and amplitude of the vulnerability of arid coasts of North Africa is alarming.

The geographical extension of the high and very-high coastal and social vulnerabilities over tens to hundreds of kilometers impose stringent multidimensional management schemes to mitigate their impacts especially in urban areas. For instance, in the Nile Delta front and prodelta deposits is ongoing armoring with seawalls and jetties along the shorelines of both cities of Alexandria and Rosette. Similarly, in the Gulf of Tunis, to protect urban areas from marine submersions, seawalls and breakwater have been installed since the severe damages caused by the storm surge of 1981. The implementation of these solutions is costly and will add additional stress to the local economies.

### Coastal vulnerability and out-migration

**Figure 6:**
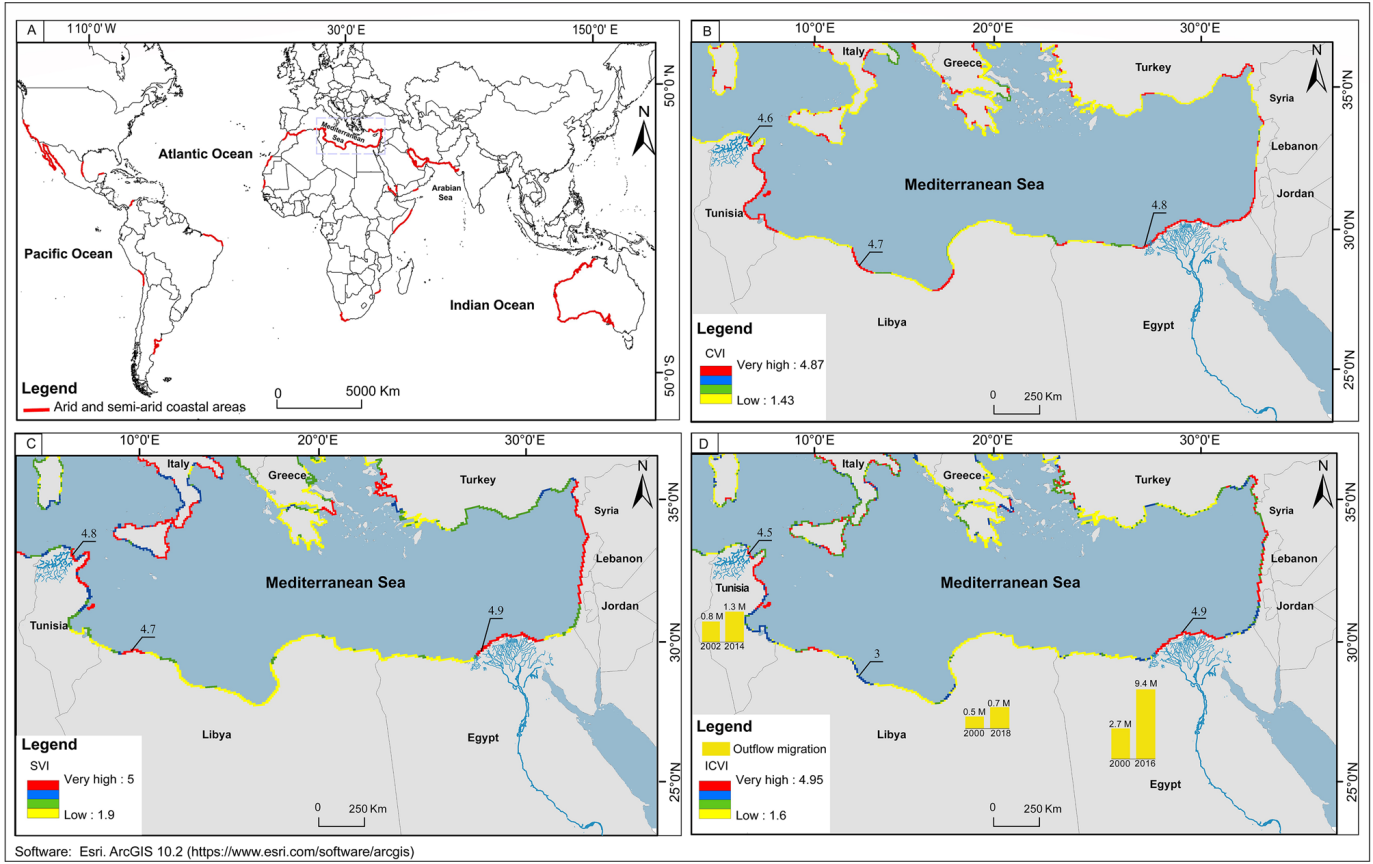
(**A**) Distribution of the world arid and semi-arid coasts; (**B**) & (**C**) Coastal and Social Vulnerabilities Indices (CVI & SVI) of the coasts of North Africa as compared to the ones of the eastern Mediterranean Basin; (**D**) Integrated Coastal Vulnerability Index (ICVI) correlated to population migratory outflows between 2000 and 2016. The arid and semi-arid coastal global geographical distribution data is from Meslier and DiRuggiero^87^.

Arid coasts represent more than 12% of the world’s coastline Fig. 6A and include several high population urban areas Fig. 6A mainly in the west coast of the United States, North Africa, the Persian Gulf, the Eastern coast of Australia as well as other areas in south America and Africa. Particularly, the arid deltaic and sandy shores of North Africa represent one of the most widespread coastal vulnerability Fig. 6B and have been associated with one of the most rapid increases in coastal urban populations of 87% and 62.6% over the last decades for both the Tunisian and Egyptian coasts respectively. The resulting environmental impacts of the rapid urban growth is the main driver for their high socioeconomic vulnerability Fig. 6C. The integrated coastal vulnerability is the result of both the combination of these physical and socioeconomic vulnerabilities and is the primary driver for significant increases in out-migration flow rates by 0.5 M (~ 62%) and 6.7 M (~ 248%) in Tunisia and Egypt respectively from 2000 to 2016, both factoring in a population of 19 M Fig. 6D.

In Fig. 6C we note that the highest socioeconomic vulnerability index values correlate with areas with the most urban land use and highest population growth rate. While there are naturally hazardous coastal events that increase the coastal vulnerability, their frequency, intensity and impacts are being affected by anthropogenic drivers. Moreover, Newton and Weichselgartner^32^ suggest two factors that increase coastal vulnerability. The first is the increase of Sea Surface Temperature (SST) which catalyzes storm occurrence, and the second is the continuous shore subsidence arising from over-extraction of coastal aquifers. Our results suggest that shoreline retreat resulting from anthropogenic tapping of sediment discharge to the coast is the main factor for coastal vulnerability in arid areas of North Africa, surpassing the two other factors suggested by Newton and Weichselgartner^32^.

For instance, the North African arid coasts experience severe shoreline retreat with erosion rates exceeding − 1 to − 5 m/year that accelerate salt intrusion in shallow coastal aquifers ~ 5 km inland causing degradation of near-coastal vegetation coverage by 18%^5^. In particular, the coastal aquifers in the Gulf of Tunis have been substantially overexploited in the last few decades, with a water budget deficit rate of 3.9 Mm^3^/year in 1996. Additionally, high salinity levels with total dissolved solids (TDS) greater than 4 g/l are being widely observed in WBGT, mainly related to seawater intrusion and the formation of swamps^68^.

This degradation of aquifer water quality results in a degradation of the soil quality leading to a substantial decrease in crop production^69^ that has yet to be quantified in our study area but can unambiguously be assessed from the rapid increase in food prices^70^. The extent and amplitude of these damages to the agricultural terrains as a result of the coastal vulnerabilities observed for both Tunisia and Egypt correlate with an increasing in out-migration flow^71^. It is important to recall that in both economies, agricultural activities account for the majority of the work force mostly constituted of youth. Hence degradation in agricultural terrains is a driver for youth unemployment in these arid areas and out-migration to nearby economically prosperous areas. Out-migration flows have been related to the population’s vulnerability to natural hazards, including floods, hurricanes and coastal erosion^72^^,^ the North African arid coasts are no exception to this link. Moreover, McLeman^73^ suggests that climate change will generate higher rates of out-migration. The climatic driver may be closely interwoven with other factors that it is difficult to individually deconvolve, environmental and human drivers will not only cause, but will rather amplify existing demographic trends of out-migration to urban areas^74^.

## Conclusion

Quantifying the physical and socioeconomic coastal vulnerability of arid areas of North Africa is crucial for facilitating the implementation of flood risk management and protection for highly populated urban areas, as well as agricultural terrain. Moreover, it is crucial to forecast these vulnerabilities in light of the predicted rise of sea surface temperatures, which are resulting in increased coastal flooding from severe weather events in the Mediterranean Basin^75^. To achieve this objective, we use three indicators: the Coastal Vulnerability Index (CVI), Socioeconomic Vulnerability Index (SVI) and Integrated Coastal Vulnerability Index (ICVI) to evaluate the physical and socioeconomic coastal parameters that govern the risk of coastal submersion. We use a GIS-based multi-criteria approach and the analytic hierarchy process for the distribution and assessment of the above three indices to define coastal vulnerability to sea-level rise and natural hazards along the coastal arid areas of North Africa. Based on the combined CVI and SVI values, the ICVI reveals that 20% and 27% of Mediterranean coast are characterized by high to very high vulnerability, respectively. In particular, we observe that the arid North African coasts of Tunisia, Libya and Egypt show highest risk scores of vulnerability indices of respectively 71%, 9% and 70% to sea level rise, which is expected to reach 1.11 m by 2100^15^. The areas of highest vulnerability are identified in both the Gulf of Tunis and the Nile Delta and are mainly attributed to anthropogenic factors such as damming and urbanization, correlated with the rapid increase in coastal urban populations by 87% and 62.6%, respectively, over the last decades. Lower vulnerability scores are observed along Libyan coasts with low populations and a balanced sediment budget.

We observe that the arid North African coasts of Tunisia and Egypt, with a total population of 19 M, show very high vulnerability risk scores of 71% and 70% respectively, extending along hundreds of kilometers of coastline that makes both of these areas the largest vulnerability zones in the central and eastern Mediterranean Basin. These unusually extensive high-vulnerability areas are also correlated with observed increases in net population migration rates from Tunisia and Egypt by 0.5 M (62%) and 6.7 M (248%) respectively from 2000 to 2016. The high coastal vulnerability of both the Gulf of Tunis and the Nile Delta are found to be primarily the result of anthropogenic drivers of damming and urbanization growth over the last decades rather than the effects of global warming. We suggest that the amplitude and extent of these coastal vulnerabilities are causing significant deterioration in coastal aquifers and hence in crop production, thereby compromising food security and resulting in out-migration trends. The evolution of these vulnerability maps will be crucial for building better policies for the governance of land use in coastal areas and increasing awareness of the extent of future coastal hazards.

## Methods

We adopt the approach of the Multi-Scale Coastal and Socioeconomic Vulnerability Index developed by Gornitz and Kanciruk^76^ and applied by McLaughlin and Cooper^77^. The methodology is developed for both regional and local scales and is based on three main steps: (1) selecting geological, physical and social variables and their impacts at the local scale,(2) using GIS and remote sensing (i.e. ArcGIS 10.2 and ENVI 5) to map physical and socioeconomic variables,and (3) mapping the Coastal Vulnerability Index (CVI) and the Socioeconomic Vulnerability Index (SVI) and defining the Integrated Coastal Vulnerability Index (ICVI) for the study areas.

The Coastal Vulnerability Index (CVI) is calculated using qualitative parameters (1) geomorphology,(2) coastal slope and (3) coastal elevation, and quantitative ones such as (4) shoreline retreat rate, (5) sea-level rise, (6) mean wave height and (7) mean tide range as dimensionless “risk” variables^47^. In our investigation, seven geological and physical variables mentioned above are used to calculate the CVI as listed in Table S2. These parameters are detailed in supplementary material in Section 3 (Mapping coastal vulnerability). A mesh with each element sized at 4000 m by 4000 m is used to calculate the above indices for the arid areas of the North African coast. A finer mesh with elements size of 150 m by 300 m is used for our validation site, the Gulf of Tunis study area.

For the Socioeconomic Vulnerability Index (SVI), we use the same method by combining four quantitative socioeconomic parameters: (1) population density, (2) land use, (3) road network and (4) settlement (Table S2). These parameters described in supplementary material are not exhaustive, but they are relevant for the social vulnerability status of the study area.

The weightings for CVI, SVI (as described in the supplementary material) and ICVI are calculated using the analytical hierarchical process (AHP) method^78^. This approach is used for the coastal vulnerability studies conducted by Rao et al.^79^ and Mahapatra et al.^80^.

The AHP method developed by Saaty^81^ is summarized in the Section 3.3 (Analytical hierarchical process (AHP)) of the supplementary material and is used to determine the weighing factors needed with the help of a priority matrix. First, pairwise comparisons are carried out for all variables and the matrix is completed by assigning a relative dominant value between 1 and 9 (Table S4).

## Supplementary information

Supplementary Information.
