# Supplementary material for: Alarming coastal vulnerability of the deltaic and sandy beaches of North Africa
Source: Sci Rep. 2021 Jan 27;11:2320. doi: 10.1038/s41598-020-77926-x (PMC7840745; doi:10.1038/s41598-020-77926-x)
Supplement: Supplementary file 1 — Supplementary Information. [file 41598_2020_77926_MOESM1_ESM.docx]

**Supplementary material & Auxilary data: Study sites Description and methods details**

**Alarming Coastal Vulnerability of the Arid Deltaic and Sandy Beaches of North Africa**

Abderraouf Hzami^1^, Essam Heggy^2,3^*, Oula Amrouni^4^, Gil Mahé^5^, Mohamed Maanan^6^, Saâdi Abdeljaouad^1^

^(1)^University of Tunis El Manar, Faculty of Sciences of Tunis, Tunisia

^(2)^University of Southern California, Viterbi School of Engineering, Los Angeles, CA, USA

^(3)^Jet Propulsion Laboratory, California Institute of Technology, Pasadena, CA, USA

^(4)^National Institute of Marine Sciences and Technologies, University of Carthage, Tunisia

^(5)^HydroSciences Laboratory, IRD, CNRS, University of Montpellier, Montpellier, France

^(6)^University of Nantes, Institute of Geography and Planning, Nantes, France

**Keywords**: Coastal Vulnerability, North Africa, Sea−level Rise, Shoreline Dynamic, Gulf of Tunis, Nile Delta, Out−Migration, Mediterranean Sea

**Contact author (*):** Dr. Essam Heggy, University of Southern California (USC), Viterbi School of Engineering, 3737 Watt Way, Powell Hall of Engineering, Office 502, Los Angeles, California, 90089−1112, USA, Tel: +1 818 812 8819, Fax: +1 213 740 8677, heggy@usc.edu

ORCID: [https://orcid.org/0000−0001−7476−2735](https://orcid.org/0000-0001-7476-2735)

1. **Study area**

The Mediterranean Basin coastline extends over ~46000 km (Stewart and Morhange, 2009) with a total population of 522 M in 2019, divided among 23 countries. More than half of this coastal population lives in coastal hydrological basins (Martínez−López et al., 2019). The arid North African coast (i.e., the coastlines of Tunisia, Libya and Egypt) is located in the central and eastern parts of the Mediterranean Basin between longitudes 8°38'36.46"E and 34°13'27.89"E and between latitudes 36°56'35.53"N and 31°19'42.52"N (Fig. 1A). These arid coasts extend for ~4633 km and comprise the majority of the North African shoreline. The study area is characterized by embayed beaches along the sandy coasts, mainly bordered by low−lying landforms such as sandy features, sand spits, river plain deposits and recent vegetated dunes. The North African coast is mostly covered by sandy clay and salty loam soils that were developed in a deltaic environment (e.g., Delta of Medjerda in Tunisia and the Nile Delta in Egypt) (Table S1). The sandy shores of Tunisia, Libya and Egypt are particularly vulnerable to seafront submersion due to sea−level rise, storm surges and shoreline retreats. All of these factors have significant socioeconomic implications as a consequence of rapid coastline changes over these extensive areas.

1. **Environmental settings of the North African arid coastline**

The North African coastlines have a Mediterranean climate, characterized by warm, dry summers, and mild, wet winters. The rainfall across our study area ranges between 400 to 600 mm/year. The central and eastern parts of the Mediterranean Basin include three distinguished climatic regimes: semi-arid, arid and hyper arid, which are characterized by sparse rainfalls, cold to mild winters and hot and dry summers (Radhouane, 2013). In order to provide a comprehensive understanding of the vulnerability patterns of these arid coastlines, we provide here in a short summary of the current state of knowledge on the environmental characteristics of the Tunisian, Libyan and Egyptian coastlines that are accounted for in our study.

**2.1. Tunisian coastline: primary study site**

The Tunisian coast, located in the central part of the Mediterranean Basin in northeastern Africa, is 1733 km long, of which 444 km are along islands and wetland ridges. One third of this coastline, i.e. 579 km is made of sandy shores (Fig. 1B; Table S1). Littoral deposits from Holocene shorelines outcrop parallel to the present shoreline, and are overlain by Miocene, Pliocene and Villafranchian deposits (Chakroun et al., 2009). The low-lying coastal area of the lagoons and the river valleys are covered with Pliocene and Quaternary coastal deposits. Northwestern Tunisia’s embayed beaches are composed of carbonate-cemented siliciclastic sand dunes, alluvial deposits and wadies and are intercepted by calcareous cliffs underlain by a succession of Tertiary and Quaternary sandstone. The southern coastal ridge is dominated by Quaternary alluvial coastal plains and consolidated Tyrrhenian dunes deposit (Gzam et al., 2014). The Sabkha of Ariana is a Quaternary depression bordered by normal faults of NW−SE orientation, which led to horst and graben structures.

The Tunisian coast has a Mediterranean climate in the north, semi-arid in the center and arid in the south, which make it an ideal area to validate coastal vulnerability assessment as it undergoes different types of climatic, environmental and urban stresses that are observed across other parts of the basin. Rainfall distribution in the north is over 400 mm/year, between 150 to 300 mm/year in the center and less than 150 mm/year in the south (INM, 2018). The prevailing active wind over the Tunisian coast blows from the NW/NE during the autumn and winter seasons. The active wind of the spring and summer originates from the east/SE direction. Accordingly, the prevailing active wind which dominates the induced longshore drift along the coast blows from north to south. In addition, seasonal and occasional storm surge waves gust from the SE to east direction, inducing a longshore drift from the south to east direction (Amrouni et al., 2014).

The dominant tide along the Tunisian coast is semi-diurnal (Brahim et al., 2014) with amplitudes of ~0.12 m in the Gulf of Tunis, ~0.5 m in the Gulf of Hammamet and ~1.5 m in the Gulf of Gabes (Furlani et al., 2014).

The hydrographic network is controlled by the active Medjerda River with an average annual flow of 30 m^3^/s that can reach 3500 m^3^/s during exceptionally extreme conditions (Claude et al., 1977). Thus, the deposited sediment load decreased from 1989 (50 g/l) to 1990 (10 g/l) (Soussi & Ben Mammou, 1989). Dams have been built along the Medjerda River and its tributaries (Fig. 1B). The most important dams are: Beni Mtir and Mellegue (1954), Kasseb (1966), Lakhmes (1968), Bou Hertma (1976), Siliana and Ain Dalia (1987), R’mil (1999), which totals 606 Mm^3^ in initial water storage capacity, and Sidi Salem (1981), the largest, with 814 Mm^3^ (Zahar & Benzarti, 2008). These dams heavily diminish sediment transport to the coastline.

The Tunisian coast is home to ~65% of the total country’s population. Moreover, it is home to ~90% of the total capacity for tourist accommodation, ~70% of economic activities and a great part of the irrigated agriculture are located in these coastal areas (Bounouh, 2010). The population growth rate over the last decade is estimated at 1.03%, with a development that is ~65% urban (Tunisian National Institute of Statistics: INS, 2014) (Table S1).

**2.2 Libyan coastline: First validation site**

The Libyan coastline extends for ~1900 km along the Mediterranean Basin and is home to ~85% of the total population (~6.9 M in 2019) (Table S1) and constitutes the primary urban and agricultural areas of the country (FAO, 2016).

The coast is divided into three main topographic regions according to the continental shelf, which is wide in the western part, narrower in the central part along the Gulf of Sirte and narrows further in the eastern part. The western and central Libyan coast displays a low but irregular topography due to cemented and uncemented sand dunes, while the shoreline alternates between cliffs, sand dunes and sheets, shallow lagoons and coastal sabkhas and low-lying plains (Anketell, 1989). The eastern part is primarily comprised of rocky shores, sandy seabeds, shallow and deep water (−200 m) with a considerable number of bays and submerged and partially submerged sea caves.

The Libyan coastline is primarily composed of Tyrrhenian marine deposits and the marls and limestones of the middle Miocene (Anketell, 1989). The Gulf of Sirtis composed of calcarenites, fluvio-aeolian, alluvial, wadi, and beach and sabkha deposits (Anketell and Ghellali, 1991). In the coastal region between Misratah and Ajdabiya, we observe thick calcrete deposits on the Al Hishah and Qarat Weddah Formations and conglomeratic sediments originating from the Old Wadi Terrace (Anketell, 1989).

The Mediterranean climate along this coastal ridge is characterized by dry summers and relatively wet winters. The average annual rainfall is about 56 mm and the temperatures range between over 40°C in the summer to below 10°C in the winter. The coastal areas are affected by the northern winds that blow in the summer in the eastern Mediterranean.

Wind directions are seasonally variable, blowing from SW/NW during the winter and from NE/NNE in the summer. Longshore drift along the Libyan coast is mostly driven from west to east (Algraeo & Bouaziz, 2013). The tide is mixed with microtidale regime. The tidal amplitude ranges between 0.3 and 0.5 m (Furlani et al., 2014).

There is no permanent network of flowing rivers feeding the Libyan coastline only ephemeral rivers or wadis cross in the Gulf of Sirte originating from seasonal sparse precipitation. There are several natural desert lakes made by artesian groundwater sapping, creating unique desert ecosystems and providing water supply to the local desert population (Abufayed et al., 2015), but those water bodies do not contribute the shoreline sediment transport mechanism. As the majority of urban, agricultural, and industrial activities are centered on the coast, the salinization of soils, freshwater contamination, and infrastructure damage pose great risks to development sustainability. Floods due to increased rain intensity on the coast may further increase the rate of coastal erosion and damage drainage and piping infrastructure (Bindraet al.,2015). The population growth rate over the last decade is estimated to be 1.45%, with development that is dramatically urban ~80% (Zurqani et al., 2019).

**2.3 Egyptian coastline: Second validation site**

The Egyptian coastline extends over ~1000 km along the Mediterranean Basin. This coast is characterized by three geomorphologic entities: the western desert coast along ~550 km, the Nile Delta coast along ~250 km in the middle seafront and the coast north of Sinai of about ~200 km (Hereher, 2015).

The geological characteristics of the Egyptian coastline indicate that all rocky outcrops are from Quaternary age. The Nile silt deposit characterizes large areas of the Nile Delta. The eastern parts of the Delta are composed of marsh, silt and clay evaporates. Large areas of the region are composed of Nile silt especially along western parts of the Rosetta Promontory. The western part of the Nile Delta is dominated by Pleistocene calcarenite bars (El−Hattab, 2015). Aeolian deposits forming sand dunes are the common terrain type in the eastern part of Nile Delta and Abu Qir Bay. Sabkha deposits and marsh, silt and clay evaporates characterize the central parts of Abu Qir Bay and the eastern parts of the Delta. Sands, gravels and recent coastal deposits are found along the Delta between the two branches of the River Nile. Most of western part of the study area is characterized by the Alexandria formation, which represents the calcarenite bars of Pleistocene age that form mainly from chalky, marly, are naceousoolitic beach ridges. Stabilized sand dunes spread inland except in the Rosetta region where it is close to the coast with an average height of 2−3 m (Embabi, 2017).

Egyptian coasts have a Mediterranean climate with temperatures ranging from 40°C in the summer to 10°C in the winter. Rainfall along this coast varies between 100 and 200 mm/yr. The dominant wind direction along the Nile’s Mediterranean coast comes from the north and NW directions during the winter season. In the summer, northerly winds prevail, while during spring, the predominant direction of wind varies between the north and NW, with occasional winds from the NE direction.

The prevailing wave direction is from the NW with an average wave height of 0.5 m (Frihy et al., 2010). In the winter, waves come from the north, NNW and NW directions. Longshore transport is predominantly driven by incident waves from west to east (in 62 to 65% cases). The Nile coast is characterized by a microtidal regime, where the tidal range is between 0.16 and 0.88 m (El-Fishawi, 1989). The sea-level rise along the Mediterranean coast of Egypt varies between 1.8 mm/yr to 2.8 mm/yr (Frihy et al., 2010).

The hydrographic network in Egypt is controlled by the active Nile River with a freshwater discharge of 2778 m^3^/s. Thus, the sediment discharge is about 3876 kg/s (Coleman & Huh, 2004). Dams have been built along the Nile River and its tributaries. The most important dams are: Zifta (1901), Assiut (1902), Low Aswan (1902), Esna (1908), Nag Hammadi (1930), Idfina (1950), High Aswan (1964) and Fraskour (1965) (Frihy, 2004).

About half of Egypt’s ~104 M population lives in the Nile Delta region. However, ~10% of Egypt population is concentrated in coastal areas (CAPMAS, 2013). Outside of major cities, population density in the Delta averages ~1000 people/km^2^. The coastal city of Alexandria is the largest in the Delta with an estimated population of more than 6 M. The population growth rate over the last decade is estimated to be 2%, with development that is dramatically urban ~50% (El−Mallakh,2020).

1. **Mapping coastal vulnerability**

We adopt the approach of the Multi-Scale Coastal and Socioeconomic Vulnerability Indices developed by Gornitz and Kanciruk (1989) and applied by McLaughlin and Cooper (2010). The methodology is developed for both regional and local scales and is based on three main steps: (1) selecting geological, physical and social variables and their impacts at the local scale; (2) using GIS and remote sensing (i.e. ArcGIS 10.2 and ENVI 5) to map physical and socioeconomic variables; and (3) mapping the Coastal Vulnerability Index (CVI) and the Socioeconomic Vulnerability Index (SVI) and defining the Integrated Coastal Vulnerability Index (ICVI) for the study areas. The vulnerability of a coastal area is defined as the response of the coast to sea-related natural hazards. Most of the existing studies use 5 to 10 variables as coastal geomorphology, coastal slope, coastal land use, shoreline change, tide range, wave height, sea-level rise, elevation and rock type to assess coastal vulnerability (e.g., Gornitz, 1991; Thieler and Hammer−Klose, 1999; Diez et al., 2007; Szlafsztein and Sterr, 2007; Nageswara Rao et al., 2008; Djouder et al., 2017; Tahri et al., 2017; Maanan et al., 2018).

Several approaches to assess coastal vulnerability have been developed and have brought modifications to the original index. For instance, Diez et al., (2007), Kumar et al. (2010) and Mahendra et al. (2011) used 7 to 8 parameters, while others only used 4 to 5 (Abdo, 2013; Kunte et al., 2014). Certain geomorphological parameters have also been considered more important in some studies and less in others (Gornitz et al., 1991; 1994).

The Coastal Vulnerability Index (CVI) is calculated using qualitative parameters such (1) geomorphology, (2) coastal slope and (3) coastal elevation, and quantitative ones such as (4) shoreline retreat rate, (5) sea-level rise rate, (6) mean wave height and (7) mean tide range as dimensionless “risk” variables (Gornitz, 1991). In our investigation, seven geological and physical variables mentioned above are used to calculate the CVI as listed in Table S2. A mesh with each element sized at 4000 m by 4000 m is used to calculate the above indices for the arid areas of the North African coast. A finer mesh with elements size of 150 m by 350 m is used for our validation site, the Gulf of Tunis study area.

For the Socioeconomic Vulnerability Index (SVI), we use the same method by combining four quantitative socioeconomic parameters: (1) population density, (2) land use, (3) road network and (4) settlement (Table S2). These parameters are not exhaustive, but they are relevant for the social vulnerability status of the study area.

The weightings for CVI and SVI are calculated using the Analytical Hierarchical Process (AHP) method detailed by Forman and Gass (2001). An Integrated Coastal Vulnerability Index (ICVI) is calculated by combining both the CVI and SVI.

- 1. **Vulnerability assessment on the regional scale**

The geological, physical and socioeconomic parameters are obtained from published data and international databases for the North African coast as listed in Table S3. The input data for our investigation of the coastal vulnerability has been implemented using ArcGIS 10.2. For mapping purposes, all data stored in raster format is transformed to vector format with WGS82 projection. Missing coastal fragments due of insufficient data describing the properties of the North African shores represent 45 km (1%) of the total coastline of 4633 km.

After identifying the geologic, physical and socioeconomic parameters of the entire Mediterranean coast from the source data, the variables of each factor are arranged in GIS and all parameters have been assigned corresponding numerical values from 1 (very low) to 5 (very high) for each segment of study area (Table S2).

- 1. **Vulnerability assessment on the local scale**

Tunisia is classified as the most vulnerable country in North Africa due to predictions of extreme climate change events, according to the 2020 Global Climate Risk Index (Eckstein et al., 2019). Our study site is located on the NE extremity of the coast of Tunisia, in the Gulf of Tunis between 37°10'N, 10°16'E and 36°55'N, 10°18'E. The western bay of the Gulf of Tunis is a regular coastline with a series of lagoons on its northern and middle parts, the Ghar El Melah, Kalâat El Andalous and Ariana lagoons (Fig. 1C). The coastal area is bordered by rocky cliffs: Gammarth Cape and Ferina Cape, which range in age from Pliocene to Neotyrrhenian coastal deposits. Low−lying coastal areas such as the river valleys of Medjerda and lagoon’s depression is covered by Pliocene and Quaternary coastal deposits. The morphology of the Gulf is the result of sedimentary filling from fluvial origin since the end of the Holocene transgression, coming from the Medjerda River (Pleuger et al., 2019).

The sandy beach, about 35 km long and 40 m to 200 m wide, is lined with fairly well−developed coastal dunes in the middle part of the Gulf (delta of Medjerda beach, Raoued beach) (Fig.1B). The dune ridge is unevenly widespread from south to north; its width varies between 30 to 50 m southwards of the bay, narrows down to just a few meters in the Gammarth and Raoued beaches, largest in the Medjerda Valley (120 to 400 m) and almost disappears in front of the Ghar El Melah lagoon. It reappears in the NE ridge of the harbour structure, but it does not exceed 80 m in the north. The tide is under microtidal range, with a tidal range lower than 0.1 to 0.3 m during the spring.

- 1. **Analytical hierarchical process (AHP)**

The weightings for CVI, SVI (as described in Appendix 1) and ICVI are calculated using the analytical hierarchical process (AHP) method (Forman and Gass, 2001). This approach is used for the coastal vulnerability studies conducted by Rao et al. (2008) and Mahapatra et al. (2015).

The AHP is developed by Saaty (2001) and is used to determine the weighing factors needed with the help of a priority matrix. First, pairwise comparisons are carried out for all variables and the matrix is completed by assigning a relative dominant value between 1 and 9 (Table S4).

They classify beach geomorphology and slope as more important than other parameters (elevation, mean wave height, mean tidal range and shoreline rate). Similarly, in this study geomorphology (priority = 34%) and coastal slope (priority = 31%) are considered significant factors for the WBGT coast and the Mediterranean coast. Additionally, shoreline change rate and elevation values are given an average ranking. The priority values were computed for socioeconomic variables, giving importance in this case to population density (60%) and coastal land use (25%).

The results of the ranking and pairwise comparison of physical variables and socioeconomic variables are shown in Table S5. The normalized eigenvector of the matrix is shown in Tables S6. In the final step, the average values of each of the seven rows are calculated for CVI, whereas the average values of four variables in each row are calculated for SVI. In order to metrically assess that the matrix judgments are randomly produced, a consistency index known as the consistency ratio (CR) is used for the AHP formulation presented by Saaty (1977) as below:

CR = CI/RI Eq. (1)

Where CI is the Consistency index and RI is the Random Index. The Consistency Index (CI) is determined from the matrix equation:

CI = (λ_max_– n)/(n – 1) Eq. (2)

Where λ max is the largest or principal eigen value of the matrix and n is the order of the matrix. The Random Index (RI) is defined as the average of the consistency index (Table S7). Generally, a Consistency Ratio (CR) with a value of 0.1 or less is considered as relevant. The CR obtained for both variables is less than 0.1 as shown in Table S7, indicating a high level of consistency, so they can be considered for further calculations. The consistency ratio obtained using the AHP is used for calculating the CVI and SVI (Eq. (3) and (4)).

CVI= W_p1_X_p1_ + W_p2_X_p2_ + W_p3_X_p3_ + W_p4_X_p4_ + W_p5_X_p5_ + W_p6_X_p6_ + W_p7_X_p7_ Eq. (3)

SVI =W_s1_X_s1_ + W_s2_X_s2_+ W_s3_X_s3_ + W_s4_X_s4_ Eq. (4)

Where W_pn_ and W_sn_ are the weight values of each of the physical and socioeconomic variables. Similarly, X_pn_ and X_sn_ are the vulnerability scores for of each the physical and socioeconomic variables. The four socioeconomic parameters and the seven physical ones are listed above in this section and summarized in Table S2.

Once the coastal and socioeconomic vulnerabilities indices are calculated from Eq. (3) and (4) for each point of the coastline under investigation, we finally calculate the ICVI as in Eq. (5):

ICVI = (CVI + SVI)/2 Eq. (5)

Which provide a full comprehensive vulnerability assessment of the shoreline averaging the two previous indices described in Equations 3 and 4.

We create the coastal vulnerability maps using the weights and scores calculated from analytic hierarchy process to delineate areas with the different vulnerability levels as follow: very low, low, medium, high, and very high. The pairwise approach extracts the most weighted physical and socioeconomic variables that control the vulnerability assessment.

**3.4 Shoreline dynamic**

To perform long-term shoreline monitoring, we use the Digital Shoreline Analysis System (DSAS) method detailed in Thieler et al., (2009) and implemented with datasets from Spot (1988 and 1999) and Sentinel 2B (2016), aerial photography (1974), topographic maps (1936) and marine maps (1882). The Cross-shore transects through the investigated coastlines are 1000 m long and are used to quantify the shoreline changes that occurred along the coast of the Gulf of Tunis. The statistics of the computed rate-of-change allow us to generate time-series integrated in GIS showing the temporal evolution of the shoreline’s geometries and positions over the last century. In our analysis, we use two statistical outputs from the DSAS model: (1) the Net Shoreline Movement (NSM), which provides the total reported separation between the oldest (1882) and current (2016) shoreline positions; and (2) the End Point Rate (EPR), which is the annual rate of shoreline retreat. Finally, the error in assessing shoreline change rates are quantified using the algorithms of Morgan et al. (1997) and Van der Wal and Pye (2003). For the NSM and EPR of this study, we adopt a margin of error from the signal to noise (S/N) ratio assumption by Dolan et al. (1980) and Thieler and Danforth (1994) (Table S2).

**REFERENCES**

1. Abdo, K. Assessing coastal vulnerability index to climate change: the case of Accra–Ghana. Proceedings 12th International Coastal Symposium (Plymouth, England). J. Coastal Res. Spec. Issue. No. **65**, 1892–1897 (2013).
2. Abufayed, A., Madi, L. & Radi, M. Libya State of the Water Reporting, Monitoring and Evaluation Operational Framework and Guidelines (2015).
3. Algraeo, J. & Bouaziz, S. The Wave Properties of Zuara Coast and Their Effects on the Marine Navigation, Open Journal of Marine Science, Vol. **3** No. 2, 93–102 (2013).
4. Amrouni, O., Hermassi, T., Abdeljaouad, S. & Messaoudi, S. Contribution of Grain–size Trend to Sediment of a Microtidal Beach. Case of the Gulf of Tunis Bay (Cape Ferina–Cape Gammarth, Tunisia). Research Journal of Environmental Sciences, **8**, 161–177 (2014).
5. Anketell, J. Quaternary Deposits of Northern Libya: Lithostratigraphy and Correlation. Libyan Studies, **20**, 1–29 (1989).
6. Anketell, J. M. & Ghellali, S. A. Paleogeologic map of the pre–Tertiary surface in the region of the Jifarah Plain andits implication to the structural history of the northern Libya; In: The Geology of Libya (eds), VI, 2381–2406 (1991).
7. Aviso (Archiving, Validation and Interpretation of Satellite Oceanographic data). Tide amplitude (Mediterranean Sea) (2013).
8. Bindra, S., Soul, F., Jabu, S., Allawafi, A., Belashher, A. et al. Potentials and prospects of renewables in Libya. In: Dincer I., Colpan C., Kizilkan O., Ezan M. (eds) Progress in Clean energy, volume 2. Springer, ChamBlack, R. et al. 2011. The effect of environmental change on human migration. Glob. Environ. Change **21**, S3–S11 (2015).
9. Bounouh, A. Nouvelles approches en matière de protection et de gestion du littoral en Tunisie. Méditerranée, **115**, 45–53 (2010).
10. Brahim, M., Atoui, A., Sammari, C. & Aleya, L. Surface sediment dynamics along the eastern coast of Djerba Island (Gabes Gulf, Tunisia). J. Afr. Earth Sci. **92**, 45–54 (2014).
11. CAPMAS, Central Agency for Public Mobilization and Statistics. Statistical Yearbook. http://www.capmas.gov.eg/Pages/Publications.aspx?page_id=5104 (2013).
12. Chakroun, A., Zaghbib–Turki, D., Miskovsky, J. C. & Davaud, E. Two Tyrrhenian transgressive cycles in coastaldeposits of the Cap Bon Peninsula, Tunisia. Quaternaire Revue de l'Association française pour l'étude du Quaternaire **20**, 2, 215–226 (2009).
13. Church, J. A., Clark, P. U., Cazenave, A., Gregory, J. M., Jevrejeva, S. et al. Sea Level Change. In: Climate Change 2013: The Physical Science Basis. Contribution of Working Group I to the Fifth Assessment Report of the Intergovernmental Panel on Climate Change [Stocker, T.F., D. Qin, G.–K., Plattner, M. Tignor, S.K., Allen, J., Boschung, A., Nauels, Y., Xia, V., Bex & P.M. Midgley (eds.)]. Cambridge University Press, Cambridge, United Kingdom and New York, NY, USA. (Chapter of the IPCC report related to sea–level rise) (2013).
14. Claude, J., Francillon, G. & Loyer, J. Y. Les alluvions déposées par l'Oued Medjerda lors de la crue exceptionnelle de mars 1973. Cahiers ORSTOM, Ser. Hydrol. **14**, 37–109 (1977).
15. Coleman, J. M. & Huh, O. K. Major world deltas: A perspective from space. [Report] Coastal Studies Institute and Department of Oceanography and Coastal Sciences, Louisiana State University, Baton Rouge, Louisiana (2004).
16. Diez, P. G., Perillo, G. M. E. & Piccolo, M. C. Vulnerability to sea–level rise on the coast of the Buenos Aires Province. Journal of Coastal Research, 23, Issue **1**, 119 – 126 (2007).
17. Djouder, F. & Boutiba, M. Vulnerability assessment of coastal areas to sea–level rise from the physical and socioeconomic parameters: case of the Gulf Coast of Bejaia, Algeria. Arabian Journal of Geosciences, 10, no. **14**, 205–223 (2017).
18. Dolan, R., Hayden, B., May, P. & May, S. The reliability of shoreline change measurements from aerial photographs. Journal of Shore and Beach, **48**, 22–29 (1980).
19. Eckstein, D., Künzel, V. & Schäfer, L. Global Climate Risk Index 2020: Who Suffers Most from Extreme Weather Events? Weather‐Related Loss in 2018 and 1999 to 2018; Germanwatch Nord‐Süd Initiative e.V: Bonn, Germany, 42 (2019).
20. El–Fishawi, N. M. Coastal erosion in relation to sea level changes, subsidence and river discharge, Nile Delta coast. Acta Mineral Petrogr, **30**, 161–171 (1989).
21. El–Hattab, M. M. Improving Coastal Vulnerability Index of the Nile Delta Coastal Zone, Egypt. J Earth Sci Clim Change, **6**, 293 (2015).
22. El–Mallakh, N. Internet Job Search, Employment, and Wage Growth: Evidence from the Arab Republic of Egypt (English). Policy Research working paper; no. WPS 9196. Washington, D.C.: World Bank Group (2020).
23. El Sayed, A., Frihy, O. E. & Deabes, E. A. Alexandria–Nile Delta coast, Egypt: update and future projection of relative sea–level rise. Environ Earth Sci, **61**, 253–273 (2010).
24. ESA (European Space Agency). Climate Change Initiative Land Cover 300 m. http://maps.elie.ucl.ac.be/CCI/viewer/ (2015).
25. Embabi, N. S. Landscapes and Landforms of Egypt: Landforms and Evolution. Springer, World Geomorphological Landscapes (2017).
26. ESPON (European Observation Network for Territorial Development and Cohesion). The ESPON Database project. https://www.espon.eu/tools–maps/espon–2013–database (2013).
27. Esteves, L. S. & Finkl, C.W. The problem of critically eroded areas (CEA): an evaluation of Florida beaches. J. Coastal Res., SI **26**, 11−18 (1998).
28. Eurostat (European Statistical Office). Population and social conditions; Statistics in focus 38/2011. https://ec.europa.eu/eurostat/web/products–statistics–in–focus/–/KS–SF–11–038 (2011).
29. FAO. AQUASTAT Country Profile – Libya. Food and Agriculture Organization of the United Nations (FAO). Rome, Italy. http://www.fao.org/3/i9803en/I9803EN.pdf (2016).
30. Forman, E. H. & Gass, S. I. The analytic hierarchy process an exposition. Operations Research, **49**, 469–486 (2001).
31. Frihy, O. Sea–level rise and shoreline retreat of the Nile Delta promontories Egypt. Nat. Hazards, **5** (1), 65–81(2004).
32. Frihy, O. E., Deabes, E. A., Shereet, S. M. & Abdalla, F. A. Alexandria–Nile Delta coast of Egypt: update and future projection of relative sea–level rise. J Environ Earth Sci, **61**, 1866–6299 (2010).
33. Furlani, S., Pappalardo, M., Gómez–Pujol, L. & Chelli, A. Geological Society, London, Memoirs, **40**, 89–123, 2014 (2014).
34. Gornitz, V. Global coastal hazards from future sea–level rise. Palaeogeogr. Palaeoclimatol. Palaeoecol, **89**, 379–398 (1991).
35. Gornitz, V. & Kanciruk, P. Assessment of global coastal hazards from sea–level rise. Proceedings of the 6th Symposium on Coastal and Ocean management, ASCE, July, 11–14 (1989).
36. Gornitz, V., White, T. W. & Cushman, R. M. Vulnerability of the U.S. to future sea–level rise. Coastal zone 91. American Society of Civil Engineers, New York, 2354–2368 (1991).
37. Gornitz, V. M., Daniels, R. C., White, T. W. & Birdwell, K. R. The development of a coastal vulnerability assessment database, Vulnerability to sea–level rise in the U.S. southeast. Journal of Coastal Research, Special Issue, **12**, 327–338 (1994).
38. Gzam, M., ElMejdoub, N., Boussetta, S. & Younes J. Genesis and evolution of a beach–ridge plain reflecting relative sea–level rise: a case study from trab El Makhadha gulf of Gabes, southeastern Tunisia. Journal of Sedimentary Research, **84**, 183–191 (2014).
39. Hereher, M. E. Coastal vulnerability assessment for Egypt's Mediterranean coast, Geomatics, Natural Hazards and Risk, **6**:4, 342–355 (2015).
40. INM. Tunisian National Institute of Meteorology., INM, Data–base (2018).
41. INS. Tunisian National Institute of Statistics. Recensement général de la population et de l’habitat en Tunisie; Rapport préliminaire, 10– 80 (2014).
42. Kerambrun, P. Coastal lagoons along the southern Mediterranean coast (Algeria, Egypt, Libya, Morocco, and Tunisia). Unesco reports in Marine Science, 34. Paris, France, Unesco (1986).
43. Kumar, T.S., Mahendra, R.S., Nayak, S., Radhakrishnan, K. & Sahu, K. C. Coastal vulnerability assessment for Orissa State, East Coast of India. Journal of Coastal Research, **26**, 523–534 (2010).
44. Kunte, P. D., Jauhari, N., Mehrotra, U., Mahender, K., Hursthouse, A. S. & Gagnon, A. S. Multi–hazards coastal vulnerability assessment of Goa, India, using geospatial techniques. Ocean. Coast. Manag., **95**, 264–281(2014).
45. LEGOS–GRGS–CNES. Laboratoire d’Etudes en Géophysique et Océanographie Spatiales – France Groupe de Recherche en Géodésie Spatiale – France. CNES. Centre National d’Etudes Spatiales, France. http://www.legos.obs–mip.fr/ (2010).
46. Ludwig, W., Bouwman, A. F., Dumont, E. & Lespinas, F. Water and nutrient fluxes from major Mediterranean and Black Sea rivers: Past and future trends and their implications for the basin‐scale budgets, Global Biogeochem. Cycles, **24**, GB0A13 (2010).
47. Luijendijk, A., Hagenaars, G., Ranasinghe, R. et al. Author Correction: The State of the World’s Beaches. Sci Rep, **8**, 11381 (2018).
48. Maanan, M., Maanan, M., Rueff, H., Adouk, N., Zourarah, B. et al. Assess the human and environmental vulnerability for coastal hazard by using a multi–criteria decision analysis. Human and Ecological Risk Assessment: An International Journal, **24** (6), 1642–1658 (2018).
49. Mahapatra, M., Ramakrishnan, R. & Rajawat, A. Coastal vulnerability assessment using analytical hierarchical process for South Gujarat coast, India," Natural Hazards. Journal of the International Society for the Prevention and Mitigation of Natural Hazards, Springer; International Society for the Prevention and Mitigation of Natural Hazards, vol. **76** (1), 139–159 (2015).
50. Mahendra, R. S., Mohanty, P. C., Bisoyi, C., Kumar, T. S. & Nayak, S. Assessment and management of coastal multi–hazard vulnerability along the Cuddalore–Villupuram, east coast of India using geospatial techniques, Ocean Coast. Manage, **54**, 302–311 (2011).
51. Martínez–López, J., Bergillos, R. J., Bonet, F. J. & Vente, J. Connecting research infrastructures, scientific and sectorial networks to support integrated management of Mediterranean coastal and rural areas. Environ. Res. Lett. **14**, 115001 (2019).
52. McLaughlin, S. & Cooper J. A. G. A multi–scale coastal vulnerability index: A tool for coastal managers? Environmental Hazards, Volume **9**, Number 3, 2010, 233–248(2010).
53. McLaughlin, S., McKenna, J. & Cooper, J.A. G. Socio–economic data in coastal vulnerability indices: constraints and opportunities. J. Coast. Res., **36**, 487–497 (2002).
54. Morgan, A., Larson, R. & Gorman, L. Monitoring the coastal environment: part III: geophysical research methods. Journal of Coastal Research, **13**, 1064–1085 (1997).
55. Nageswara Rao, K., Subraelu, P., Venkateswara Rao, T., HemaMalini, B., Ratheesh, R. et al. Sea–level rise and coastal vulnerability: an assessment of Andhra Pradesh coast, India through remote sensing and GIS. Journal of Coastal Conservation, **12**, 195–207 (2008).
56. Oliveau, S. & Doignon, Y. Ever closer to the water. Recent developments in Mediterranean settlement patterns. South–East European Journal of Political Science, II (3) (2014).
57. Oueslati, A. Les côtes de la Tunisie ; Géomorphologie et Environnement et aptitudes à l’Aménagement, Publications de la faculté des Sciences Humaines et Sociales, ISBN 10 : 9973900650. Tunis, 387 (1993).
58. Pirazzoli, P. A. Secular trends of relative sea level (RSL) changes indicated by tide–gauge records, J. Coast. Res, **1**, 1–26 (1986).
59. Pirazzoli, P. A. Present and near future global sea level changes, Paleogeography, Paleoclimatology, Paleoecology, **75**, 241–258 (1989).
60. Pleuger, E., Goiran, J. P. h., Mazzini, I., Delile, H., Abichou, A. et al. Paleogeographical et paleoenvirenmental reconstitution of the Medjerda delta (Tunisia) during the Holocene. Quaternary Science Reviews, **220**, 178–263 (2019).
61. Radhouane, L. Climate change impacts on NorthAfrican countries and on some Tunisian economic sectors, J. Agric. Environ. Int. Dev. (JAEID), vol. **107**, no. 1, 101–113 (2013).
62. Rao, H., Korczykowski, M., Pluta, J., Hoang, A. & Detre, J. A. Neural correlates of voluntary and involuntary risk taking in the human brain: an FMRI Study of the Balloon Analog Risk Task (BART). NeuroImage, **42**, 902–910 (2008).
63. Saaty, T. L. A scaling method for priorities in hierarchical structures. J. Math. Psychol, **15**, 234–281 (1977).
64. Saaty, T. L. Fundamentals of decision–making and priority theory, Pittsburgh, RWS Publications. ISBN No–0962031763 (2001).
65. Saaty, T.L. & Vargas, L. G. Prediction, Projection and Forecasting, Boston: Kluwer. Academic (1991).
66. Soussi, N. & Ben Mammou, A. Les apports solides en suspension de la Medjerda–Tunisie. 2ème congrès de sédimentologie Paris (1989).
67. Szlafsztein, C. & Sterr, H. A. GIS–based vulnerability assessment of coastal natural hazards, state of Para, Brazil. J. Coastal Res., **11**, 53–66 (2007).
68. Stewart, I. S. & Morhange, C. Coastal geomorphology and sea–level change. In: Woodward, J.C. (Ed.). The Physical Geography of the Mediterranean Basin. Oxford, University Press, Oxford, 385–413 (2009).
69. Tahri, M., Maanan, M. Hakdaoui, M. Using Fuzzy Analytic Hierarchy Process multicriteria and Geographical information system for coastal vulnerability analysis in Morocco: The case of Mohammedia. Prog. Phys. Geogr (2017).
70. Thieler, E. R. & Danforth, W. W. Historical shoreline mapping (II): application of the Digital Shoreline Mapping and Analysis Systems (DSMS/DSAS) to shoreline change mapping in Puerto Rico. J. Coastal Res, **10**, 600–620 (1994).
71. Thieler, E. R. & Hammar–Klose, E. S. National assessment of coastal vulnerability to future sea–level rise: preliminary results for the U.S. Atlantic coast. U.S. Geological Survey, Open–File Report, 99–593 (1999).
72. Thieler, E.R., Himmelstoss, E.A., Zichichi, J. L. & Ergul, A. Digital shoreline analysis system (DSAS) version 4.0. An ArcGIS extension for calculating shoreline change: U.S. Geological Survey Open–File Report 2008–1278 (2009).
73. Van der Wal, D. & Pye, K. The use of historical bathymetric charts in a GIS to assess morphological change in estuaries. The Geographical Journal, **169**, 21–31 (2003).
74. UNEP/MAP. Mediterranean Action Plan. State of the Mediterranean Marine and Coastal Environment, highlights for policy makers. UNEP/MAP – Barcelona Convention, Athens. https://wedocs.unep.org/bitstream/handle/20.500.11822/375/unepmap_soehighlights_2012_eng.pdf?sequence=3&isAllowed=y (2012).
75. United Nations. United Nations population estimates and projections of major Urban Agglomerations. Revision of World Urbanization Prospects which are consistent with the size of the total population of each country as estimated or projected in the 2017 Revision of World Population Prospects. https://population.un.org/wup/(2018).
76. Zahar, Y. & Benzarti, Z. Impact du barrage de Sidi Salem sur les risques d’inondation dans la basse vallée de la Mejerda. In: Unité de recherche GREVACHOT (Ed.), Atlas de l’eau en Tunisie. FSHS de Tunis, 144–149 (2008).
77. Zaïbi, C., Kamoun, F., Viehberg, F., Carbonel, P., Jedoui, Y., Abida, A. & Fontugny, M. Impact of relative sea level and extreme climate events on the Southern Skhira coastline (Gulf of Gabes, Tunisia) during Holocene times: ostracodes and foraminifera associations response. J. Afr. Earth Sci., **118**, 120–136 (2016).
78. Zurqani, H. A., Mikhailova, E. A., Post, C. J., Schlautman, M. A. & Elhawej, A. R. A Review of Libyan Soil Databases for Use within an Ecosystem Services Framework. Land*,***8***,* 82 (2019).

**CaptionS and tables**


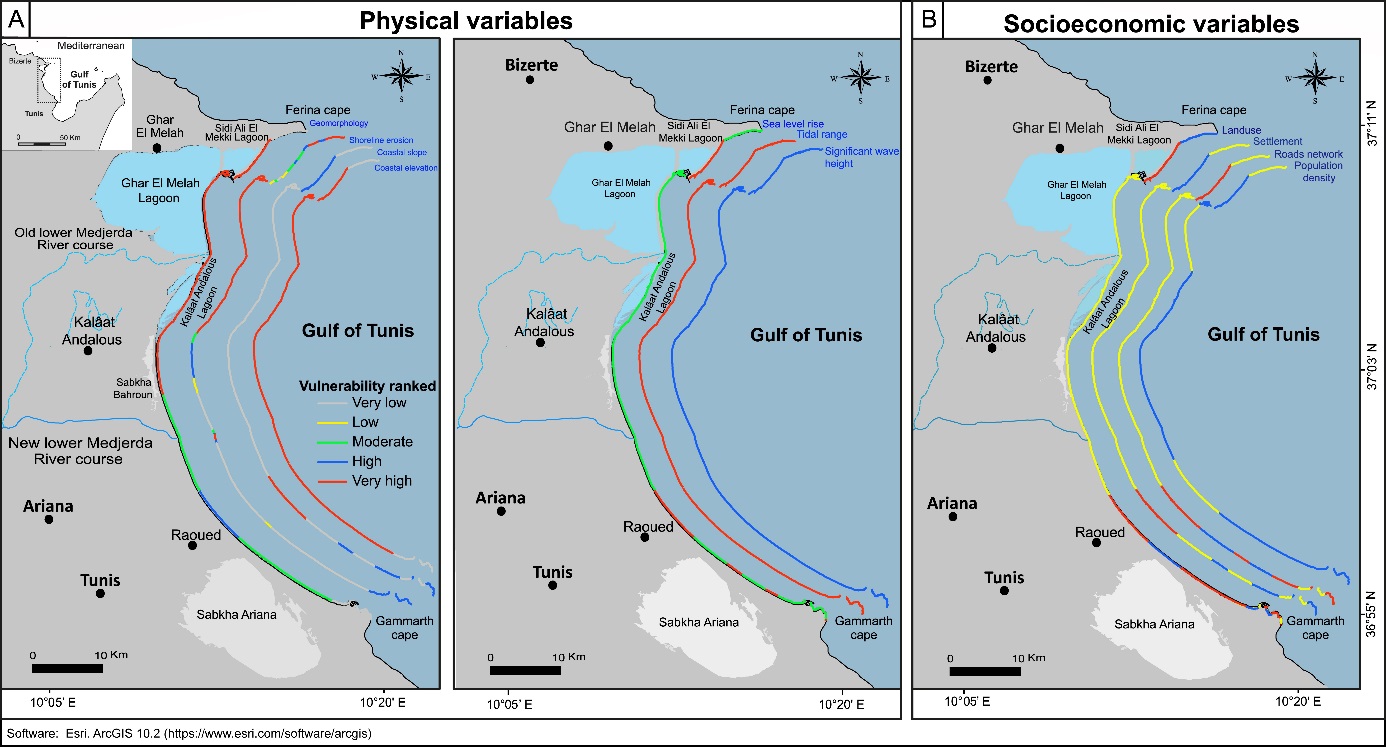


**Supplementary data: Fig. S1**: Mapping vulnerability hazard variables established for A) the Coastal Vulnerability Index CVI and B) Socioeconomic Vulnerability Index SVI along primary study coast of the western bay of the Gulf of Tunis, Mediterranean.


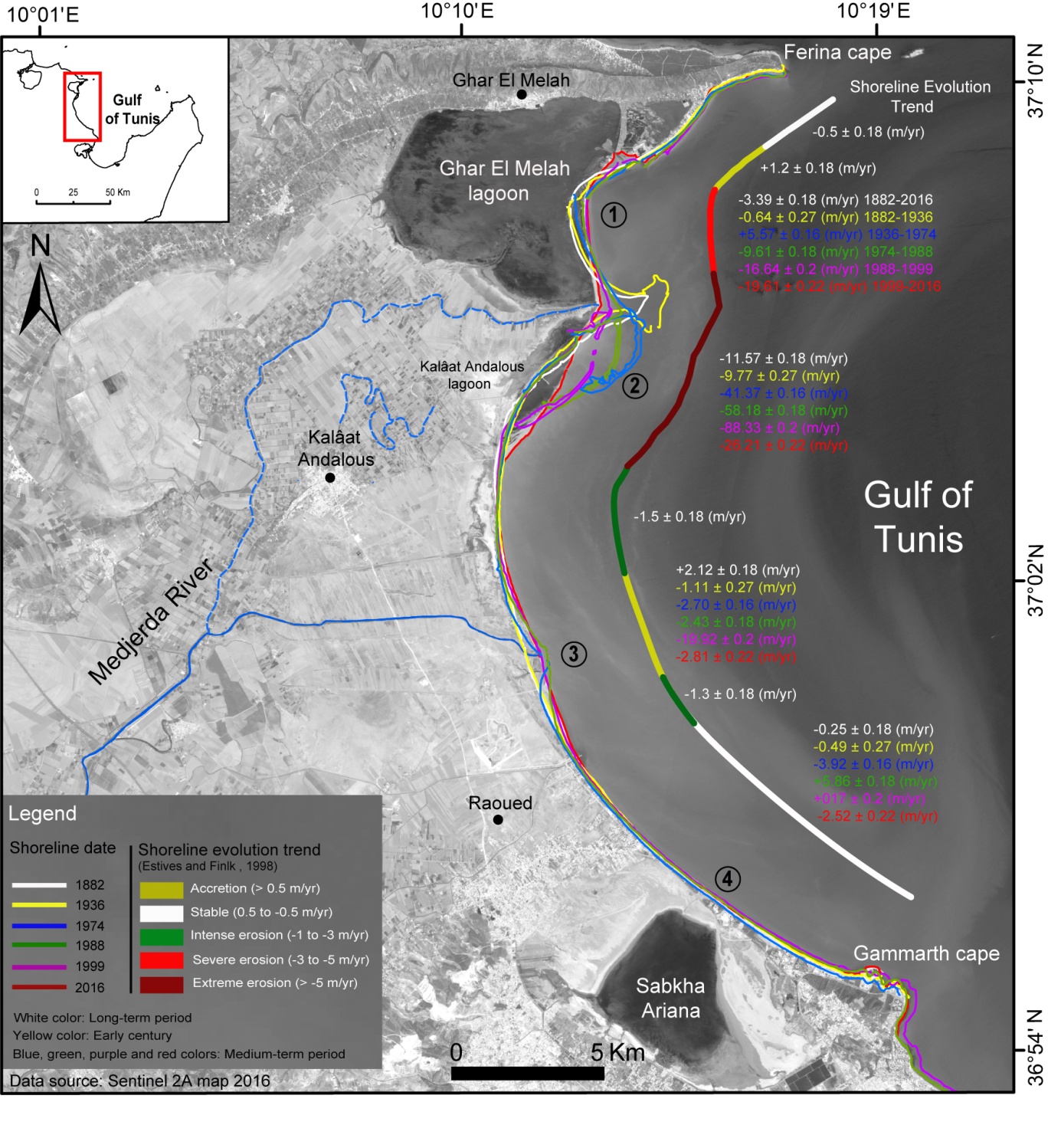


**Supplementary data: Fig. S2**: Map synthesizing the shoreline movement trend at the beaches of the Gulf of Tunis during the 1882−2016 periods. The white, yellow, green, purple and red colors correspond to the detailed End Point Rate movement (EPR). Shoreline movement is calculated by DSAS model from bathymetric and topographic maps (1882−1936 and 1974) and the SPOT and Sentinel photogrammetric orbital scene’s (1988−1999 and 2016).We classify the different observed erosion rates according to the classes of shoreline trends established by Esteves and Finkl (1998).

**Supplementary data: Table S1**: Characteristic, physical and social parameters of the microtidal (< 2 m) coastal areas.

^(1)^UNEP/MAP 2012;^(2)^Kerambrun, 1986;^(3)^Hereher, 2015; ^(4)^Ludwig, 2010;^(5)^El Sayed et al., 2010;^(6)^Zaïbi et al., 2016,^(7)^Church et al., 2013;^(8)^Pirazolli, 1986; ^(9)^Pirazolli, 1989;^(10)^ INS, 2014,^(11)^ FAO, 2016;^(12)^El−Mallakh, 2020; ^(13)^Oliveau et al., 2014; ^(14)^ United Nation, 2018.

|  | **Total length (km)**  **^(1)(2)(3)^** | **Geomorphology classification**  **Percentage (%)** | **Beach berm gradient (slope)** | **Major river network, Debit flow (m^3^/s)^(4)^** | **Mean annual river discharge**  **(m^3^/s)^(4)^** | **Breaking height**  **wave H_b_ (m)^(3)^** | **Subsidence**  **(mm/yr)**  **^(5)(6)^** | **Relative sea level rise change**  **(mm/yr)**  **^(7)(8) (9)^** | **Population Number**  **(Million)**  **^(1)(10)(11)(12)^** | **Population density (Hab/km^2^)**  **^(13) (10)(14)(12)^** | **Urban population**  **(Millions)**  **Percentage (%)**  **^(10)(12)^** |
| --- | --- | --- | --- | --- | --- | --- | --- | --- | --- | --- | --- |
| **Mediterranean coasts** | 46000  Maritime shorelines: 27000  Island shorelines: 19000 | - Sandy beach (23.8%) - River deltas, estuaries and soft sedimentary strands (3.6%) - Artificial structures and artificial frontage (9.8%) - Soft rock shores (23.2%) - Hard rock shores (30.05%) | <10° | - Moulouya, Morrocco: 50 - Chelif, Algeria :49 - Medjerda, Tunisia: 30 - Nile, Egypt: 2830 - Ebre, Spain : 426 - Rhône, France : 1700 - Tibre, Italy: 267 | 10000 | 1 to 5 | − | +1.5 to + 3.2 | - 276 in 1970 - 412 in 2000 - 466 in 2010 | 100 | 152 in 1970  315 in 2010  1.9% |
| **NorthAfrican coasts** | Tunisia  2290  Maritime shorelines: 1733  Island shorelines: 450 | - Sandy beach (33%) - River deltas, estuaries, sabkhas and marshes (18%) - Cliffs and rocky shores (31%) - Artificial structures and other frontage (48%) | 1– 5° | Medjerda : 30 | 1000 | Winter & Spring:  W to NW: H_b_= 0.7– 6  N to NE: H_b_= 0.9– 1.45  Summer & Autumn:  E: H_b_= 1.5– 2.45  SE: H_b_= 1.95– 3 | 1992−2000  Ghar El Melah: 10 | +1.3 in Tunis;  2 in Sousse +4.3 in Sfax | 10.9 in 2014 | 1305 | 65 |
|  | Libya  2000 | - Sandy beach (34.25%) - Cliffs and rocky shores 1,290 km (64.5%) - Lagoon and Sabkhas (Ain Azzarga) 25 km (1.25%) | 1– 35° | No rivers | − | Winter: SW– W  Spring: N– E  Summer & Autumn: N−NE | Unavailable | Unavailable | 6.9 in 2019 | 200 | 50 |
|  | Egypt  1000 | - Deltaic and sandy ridges (45%) - Coastal lakes and lagoons (15%) - Low cliff and rocky shores (30%) | 2– 6.5° | Nile : 2830 | 3000 | Spring & Summer:  NNW & NE:  H_b_= 0.56 – 0.92  Winter:  NNW– WNW:  H_s_ = 1.12– 1.91  H_b_ = 1.5 m | Rosetta : 2.5  Damietta: 3.5 | 1969−2008  Rosetta : 4.9  1990−2007  Damietta: 2.9 | 104 in 2020 | 1500 | 80 |

**Supplementary data: Table S2**. Ranges of the Coastal Vulnerability Index, CVI and Socioeconomic Vulnerability Index SVI (Thieler and Hammar−Klose, 1999; Gornitz et al., 1991, 1994).

| **Variables** | | **Score** | | | | |
| --- | --- | --- | --- | --- | --- | --- |
|  |  | **Very low 1** | **Low 2** | **Moderate 3** | **High 4** | **Very high 5** |
| **Physical** | **Geomorphology** | Rocky, cliff, headland coasts | Medium cliffs, indented coasts | Low cliffs, alluvial plains | Cobble beaches, lagoons | Barrier, beaches, mangroves, mudflats, deltas |
|  | **Coastal slope (%)** | > 12 | 12– 9 | 9– 6 | 6– 3 | < 3 |
|  | **Coastal elevation (m)** | > 30 | > 20 and ≤ 30 | > 10 and ≤ 20 | > 5 and ≤ 10 | ≥ 0 and ≤ 5 |
|  | **Sea rise level rate (mm/yr)** | < 1.8 | 1.8– 2.5 | 2.5– 3 | 3– 3.4 | >3.4 |
|  | **Shoreline**  **rates (m/yr)** | > +2.0 | +1.0 to +2.0 | −1.0 to +1.0 | −2.0 to−1.0 | <− 2.0 |
|  | **Tidal range (m)** | > 6  Macrotidal | 6– 4  Mesotidal | 4– 2  Mesotidal | 2– 1  Microtidal | < 1  Microtidal |
|  | **Significant wave height (m)** | < 0.55 | 0.55 – 0.8 | 0.85– 1.05 | 1.05– 1.25 | > 1.25 |
| **Socioeconomic** | **Population density (SQ/km)** | <100 | 100– 200 | 200– 400 | 400– 600 | >600 |
|  | **Coastal land use** | Water bodies, marsh/bog and moor, sparsely vegetated areas | Bare land | Water/wetland, grassland | Forest, farmland | Urban space |
|  | **Road network** | > 1500 | 1000–1500 | 500– 1000 | 250– 500 | <250 |
|  | **Settlement** | No settlement | Village | Small town | Large town | City |

**Supplementary data: Table S3.** Data sources used of physical and socioeconomic variables.

| **Variable** | **Description** | **Unit** | **Data source** | | |
| --- | --- | --- | --- | --- | --- |
|  |  |  | North Africa (Tunisia, Libya and Egypt) | | Gulf of Tunis  Tunisia |
| **Geomorphology** | The landform type reflects the response of coastal deposits to sea−level rise, since different types of landforms offer various degrees of resistance to erosion (Thieler and Hammer−Klose, 1999). | Classes | Furlani et al., 2014 | | Oueslati, 1993 |
| **Coastal slope** | Average topographic slope. | % | SRTM, GIS | | |
| **Coastal elevation** | Represents the surface within a class of elevation. | m | DEM, GIS | | |
| **Shoreline rate** | Rate of shoreline movement | m | Luijendijk et al., 2018 | DSAS model  Thieler et al., 2009 | |
| **Sea level rise** | Average of the Sea level increased in one−year time. | mm/yr | LEGOS−GRGS−CNES, 2010 | | |
| **Tidal range** | Vertical difference between the highest high tide and the lowest low tide (Kumar et al., 2010). | m | Satellite Aviso, 2013 | | |
| **Significant wave height** | Average height of the highest one−third in a wave spectrum for a given period of time at sea. | m | KNMI/ERA  (1972−2001) | | |
| **Land use** | The LC map from 2016 is a global land cover map at 20 m spatial resolution. | Classes | ESA, 2015 | SPOT 5 (2012)  Sentinel 2A | |
| **Population density** | The average number of people who live on each Km² of land area. | hab/km² | Eurostat, 2011 | INS, 2014 | |
| **Road network** | The road network occupies a defined space, has defined widths, and the costs of protection by relocating them are very high (McLaughlin et al., 2002). | Classes | Espon data base, 2013 | SPOT 5 (2012)  Sentinel 2A | |
| **Settlement** | Coastal cities are supposed to be more vulnerable compared to villages. | Classes |  |  |  |

**Supplementary data: Table S4.** Evaluation scale used in pairwise comparisons (Saaty, 2001).

| **Intensity of**  **importance** | **Definition** | **Explanation** |
| --- | --- | --- |
| 1 | Equal importance | Two factors contribute equally to the objective. |
| 3 | Somewhat more important | Experience and judgment slightly favor one over the other. |
| 5 | Much more important | Experience and judgment strongly favor one over the other. |
| 7 | Very much more important | Experience and judgment very strongly favor one over the other. Its importance is demonstrated in practice. |
| 9 | Absolutely more important | The evidence favoring one over the other is of the highest possible validity. |
| 2, 4, 6 and 8 | Intermediate values | When compromise is needed. |

**Supplementary data: Table S5.** Pairwise comparison matrix of physical and socioeconomic variables.

| **Geological and physical variables** | **Geomorphology** | **Coastal slope (%)** | **Coastal elevation (m)** | **Shoreline rate**  **(m/yr)** | **Sea level rise rate**  **(mm/yr)** | **Tidal range**  **(m)** | **Significant wave height (m)** |
| --- | --- | --- | --- | --- | --- | --- | --- |
| **Geomorphology** | 1 | 2.00 | 3.00 | 5.00 | 6.00 | 9.00 | 8.00 |
| **Coastal slope** | 0.50 | 1 | 4.00 | 6.00 | 7.00 | 9.00 | 9.00 |
| **Coastal elevation** | 0.33 | 0.25 | 1 | 3.00 | 4.00 | 7.00 | 5.00 |
| **Shoreline rate** | 0.20 | 0.17 | 0.33 | 1 | 3.00 | 5.00 | 4.00 |
| **Sea level rise rate** | 0.17 | 0.14 | 0.25 | 0.33 | 1 | 3.00 | 3.00 |
| **Tidal range** | 0.11 | 0.11 | 0.14 | 0.20 | 0.33 | 1 | 2.00 |
| **Significant wave height** | 0.13 | 0.11 | 0.20 | 0.25 | 0.33 | 0.50 | 1 |
| **Column total** | 2.44 | 3.78 | 8.93 | 15.78 | 21.67 | 34.50 | 32.00 |

| **Socioeconomic variables** | **Population density** | **Coastal land use** | **Road network** | **Settlement** |
| --- | --- | --- | --- | --- |
| **Population density** | 1 | 4 | 8 | 7 |
| **Coastal land use** | 0.25 | 1 | 4 | 6 |
| **Road network** | 0.13 | 0.25 | 1 | 3 |
| **Settlement** | 0.14 | 0.17 | 0.33 | 1 |
| **Column total** | 1.52 | 5.42 | 13.33 | 17 |

| **Geological and physical variables** | **Variables** | **Geo−morphology** | **Coastal slope** | **Coastal elevation** | **Shoreline rate** | **Sea level rise rate** | **Tidal range**  **(m)** | **Significant wave height (m)** | **Sum** | **Priority vector (X)** | **Percent (%)** |
| --- | --- | --- | --- | --- | --- | --- | --- | --- | --- | --- | --- |
|  | Geo−morphology | 0.41 | 0.53 | 0.34 | 0.32 | 0.28 | 0.26 | 0.25 | 2.38 | 0.34 | 34 |
|  | Coastal slope | 0.21 | 0.26 | 0.45 | 0.38 | 0.32 | 0.26 | 0.28 | 2.16 | 0.31 | 31 |
|  | Coastal elevation | 0.14 | 0.07 | 0.11 | 0.19 | 0.18 | 0.20 | 0.16 | 1.05 | 0.15 | 15 |
|  | Shoreline rate | 0.08 | 0.04 | 0.04 | 0.06 | 0.14 | 0.14 | 0.13 | 0.64 | 0.09 | 9 |
|  | Sea level rise rate | 0.07 | 0.04 | 0.03 | 0.02 | 0.05 | 0.09 | 0.09 | 0.38 | 0.05 | 5 |
|  | Tidal range | 0.05 | 0.03 | 0.02 | 0.01 | 0.02 | 0.03 | 0.06 | 0.21 | 0.03 | 3 |
|  | Significant wave height | 0.05 | 0.03 | 0.02 | 0.02 | 0.02 | 0.01 | 0.03 | 0.18 | 0.03 | 3 |
|  | Total | 1.00 | 1.00 | 1.00 | 1.00 | 1.00 | 1.00 | 1.00 | 7.00 | 1.00 | 100 |

**Supplementary data: Table S6.** Normalized matrix of physical and socioeconomic variables.

| **Socioeconomic variables** | **Variables** | **Population density** | **Coastal land use** | **Road network** | **Settlement** | **Sum** | **Priority vector (X)** | **Percent (%)** |
| --- | --- | --- | --- | --- | --- | --- | --- | --- |
|  | Population density | 0.66 | 0.74 | 0.60 | 0.41 | 2.41 | 0.60 | 60 |
|  | Coastal land use | 0.16 | 0.18 | 0.30 | 0.35 | 0.99 | 0.25 | 25 |
|  | Road network | 0.09 | 0.05 | 0.08 | 0.18 | 0.4 | 0.10 | 10 |
|  | Settlement | 0.09 | 0.03 | 0.02 | 0.06 | 0.2 | 0.05 | 5 |
|  | Total | 1 | 1 | 1 | 1 | 4 | − | 100 |

**Supplementary data: Table S7.** Values of random Index RI (Saaty and Vargas., 1991); Computation of Consistency Ratio (CR); n is the order of the matrix; RI is the random index.

| **Variables** | | **Physical variables** | | | **Socioeconomic variables** | | | |
| --- | --- | --- | --- | --- | --- | --- | --- | --- |
| **Consistency Ratio** | **n** | 2 | 3 | 4 | 5 | 6 | 7 | 8 |
|  | **RI** | 0.00 | 0.52 | 0.9 | 1.12 | 1.24 | 1.32 | 1.41 |
| **Random Index** | **Max** | 7.53 | | | 4.27 | | | |
|  | **n** | 7 | | | 4 | | | |
|  | **RI** | 1.32 | | | 0.9 | | | |

| **Study site**  **(Coast lenght)** | | **Dates (yrs)** | **Max EPR (m/yr)** | **Min EPR (m/yr)** | **Average EPR (m/yr)** | **Error EPR (m/yr)** | **Max NSM**  **(m)** | **Min NSM**  **(m)** | **Average NSM (m)** | **Error NSM (m)** |
| --- | --- | --- | --- | --- | --- | --- | --- | --- | --- | --- |
| **Northern bay** | Area 1  Ghar El Melah  (5 km) | 1882−2016 | −3.39 | 4.10 | 1.43 | 0.185 | −453.63 | 548.76 | 193.25 | 24.8 |
|  |  | 1882−1936 | −0.64 | 4.51 | 1.85 | 0.27 | −34.68 | 243.60 | 99.66 | 14.6 |
|  |  | 1936−1974 | 1.78 | 5.57 | 3.82 | 0.16 | 67.53 | 211.49 | 145.19 | 6 |
|  |  | 1974−1988 | −9.61 | 20.92 | 3.96 | 0.18 | −134.47 | 292.89 | 55.52 | 2.5 |
|  |  | 1988−1999 | −16.64 | 9.40 | −2.39 | 0.2 | −183.00 | 103.38 | −26.37 | 2 |
|  |  | 1999−2016 | −19.61 | 1.92 | −5.23 | 0.22 | −333.29 | 32.61 | −88.95 | 3.7 |
|  | Area 2  Old mouth  (5 km) | 1882−2016 | −11.57 | 2.55 | −4.31 | 0.185 | −1550.86 | 342.02 | −578.6 | 24.8 |
|  |  | 1882−1936 | −9.77 | 15.32 | 2.87 | 0.27 | −527.59 | 827.27 | 154.78 | 14.6 |
|  |  | 1936−1974 | −41.37 | 3.83 | −11.61 | 0.16 | −1571.88 | 145.50 | −441.24 | 6 |
|  |  | 1974−1988 | −58.18 | 4.55 | −24.56 | 0.18 | −814.57 | 63.72 | −343.84 | 2.5 |
|  |  | 1988−1999 | −88.33 | 0.34 | −32.64 | 0.2 | −971.64 | 3.74 | −359.04 | 2 |
|  |  | 1999−2016 | −26.21 | −4.52 | −15.95 | 0.22 | −445.58 | −76.91 | −271.15 | 3.7 |
| **Central bay** | Area 3  New mouth  (5 km) | 1882−2016 | 2.12 | 3.40 | 2.63 | 0.185 | 284.34 | 455.06 | 353.66 | 24.8 |
|  |  | 1882−1936 | −1.11 | 1.46 | 0.33 | 0.27 | −59.71 | 78.58 | 17.58 | 14.6 |
|  |  | 1936−1974 | −2.70 | 16.02 | 4.33 | 0.16 | −102.43 | 608.89 | 164.64 | 6 |
|  |  | 1974−1988 | −2.43 | 27.01 | 16.72 | 0.18 | −34.07 | 378.11 | 234.122 | 2.5 |
|  |  | 1988−1999 | −19.92 | 10.02 | −5.02 | 0.2 | −219.13 | 110.22 | −55.28 | 2 |
|  |  | 1999−2016 | −2.81 | 3.61 | −0.14 | 0.22 | −47.69 | 61.42 | −2.50 | 3.7 |
| **Southern bay** | Area 4  Raoued  (5 km) | 1882−2016 | −0.25 | 0.16 | −0.05 | 0.185 | −33.58 | 22.08 | −7.55 | 24.8 |
|  |  | 1882−1936 | −0.49 | 0.21 | −0.10 | 0.27 | −26.54 | 11.48 | −5.57 | 14.6 |
|  |  | 1936−1974 | −3.92 | −2.32 | −3.13 | 0.16 | −148.95 | −88.17 | −118.82 | 6 |
|  |  | 1974−1988 | 5.86 | 10.17 | 8.43 | 0.18 | 82.07 | 142.43 | 118.04 | 2.5 |
|  |  | 1988−1999 | 0.17 | 3.39 | 2.26 | 0.2 | 1.84 | 37.34 | 24.94 | 2 |
|  |  | 1999−2016 | −2.52 | −0.30 | −1.58 | 0.22 | −42.87 | −5.03 | −26.94 | 3.7 |

**Supplementary data: Table S8:** The shoreline evolution analysis at the beaches of the Gulf of Tunis, Tunisia. The Net Shoreline Movement (NSM) and the End−Point Rate (EPR) during the 1882−2016 period. The End−Point shoreline change Rates for the Gulf of Tunis beaches clearly show the erosion−dominated shoreline trend. Negative values indicate erosion.
